# Cannabis Laws and Utilization of Medications for the Treatment of Mental Health Disorders

**DOI:** 10.1001/jamanetworkopen.2024.32021

**Published:** 2024-09-05

**Authors:** Ashley C. Bradford, Felipe Lozano-Rojas, Hailemichael Bekele Shone, W. David Bradford, Amanda J. Abraham

**Affiliations:** 1 School of Public Policy, Georgia Institute of Technology, Atlanta; 2 Department of Public Administration and Policy, University of Georgia, School of Public & International Affairs, Athens; 3 Paul H. O’Neill School of Public and Environmental Affairs, Indiana University, Bloomington

## Abstract

**Importance:**

Mental health disorders are prevalent yet undertreated health conditions in the US. Given perceptions about the potential effect of cannabis on individuals with mental health disorders, there is a need to understand the association of cannabis laws with psychotropic use.

**Objective:**

To investigate the association of medical and recreational cannabis laws and dispensary openings with the dispensing of psychotropic medications used to treat mental health disorders in the US.

**Design, Setting, and Participants:**

This cross-sectional study of 10 013 948 commercially insured patients used a synthetic control method to examine the association of cannabis policies with prescribing. Data on all patients dispensed prescriptions for each of the 5 classes of psychotropic medications from January 1, 2007, to December 31, 2020, were extracted from Optum’s deidentified Clinformatics Data Mart Database. Statistical analysis was performed from September 2022 to November 2023.

**Exposures:**

The 4 exposure variables measured were whether medical or recreational cannabis laws were in effect and whether medical or recreational cannabis dispensaries were open in each state and calendar quarter.

**Main Outcome and Measures:**

One measure of the extensive margins of dispensing and 2 measures of the intensive margins of dispensing were constructed for 5 medication classes (benzodiazepines, antidepressants, antipsychotics, barbiturates, and sleep medications).

**Results:**

The primary sample (the benzodiazepine sample) included 3 848 721 patients (mean [SD] age, 46.1 [11.4] years; 65.4% women; 53.7% aged 35-54 years). Medical cannabis laws were associated with a 12.4% reduction in the benzodiazepine fill rate (average treatment effect on the treated [ATT], –27.4; 95% CI, –14.7 to 12.0; *P* = .001), recreational cannabis laws were associated with a 15.2% reduction in the fill rate (ATT, −32.5; 95% CI, −24.4 to 20.1; *P* = .02), and medical cannabis laws were associated with a 1.3% reduction in the mean number of benzodiazepine fills per patient (ATT, –0.02; 95% CI, −0.02 to 0.02; *P* = .04). Medical dispensaries were associated with a 3.9% reduction in mean days’ supply per benzodiazepine fill (ATT, −1.7; 95% CI, −0.8 to 0.6; *P* = .001), while recreational dispensaries were associated with a 6.2% reduction (ATT, −2.4; 95% CI, −1.0 to 0.9; *P* < .001). Medical cannabis laws were associated with a 3.8% increase in antidepressant fills (ATT, 27.2; 95% CI, −33.5 to 26.9; *P* = .048), and medical dispensaries were associated with an 8.8% increase (ATT, 50.7; 95% CI, −32.3 to 28.4; *P* = .004). The mean number of antipsychotic medication fills per patient increased by 2.5% (ATT, 0.06; 95% CI, −0.04 to 0.05; *P* = .02) after medical cannabis laws and by 2.5% (ATT, 0.06; 95% CI, −0.04 to 0.04; *P* = .02) after medical dispensary openings. Findings for the other drug classes showed substantial heterogeneity by state and direction of association.

**Conclusions and Relevance:**

This cross-sectional study of commercially insured patients suggests that there may have been meaningful heterogeneous associations between cannabis policy and state and between cannabis policy and drug class (eg, decreases in dispensing of benzodiazepines but increases in dispensing of antidepressants and antipsychotics). This finding suggests additional clinical research is needed to understand the association between cannabis use and mental health. The results have implications for patient substance use and mental health–related outcomes.

## Key Points

**Question:**

Is access to cannabis, via medical or recreational legalization, associated with changes in dispensing of prescription medications to treat mental health disorders in a commercially insured population?

**Findings:**

This cross-sectional study of 9 438 716 commercially insured patients found statistically significant reductions in benzodiazepine dispensing after increases in both medical and recreational cannabis access. However, evidence suggests increases in other types of psychotropic dispensing.

**Meaning:**

This study suggests that cannabis laws may be significantly associated with the population-level use of prescription drugs to treat mental health disorders, although the associations vary by drug class and state.

## Teaser

This cross-sectional study investigates the association of medical and recreational legalization of cannabis and dispensary openings with the dispensing of psychotropic medications used to treat mental health disorders in the US.

## Introduction

Mental health disorders (MHDs) are both common and costly health conditions in the US. In 2021, 22.8% of adults (57.8 million) reported having at least 1 MHD.^1^ Overall, 47.4% of individuals in the US reported receiving a diagnosis for an MHD at some point in their lives.^2^ Mental health disorders are costly for society, amounting to approximately $201 billion in 2013 alone.^3^ Despite this, among individuals reporting at least 1 MHD, only 65.4% received any treatment within the past year.^1^

Available treatment options for MHDs continuously evolve, and 1 recent change is the emergence of cannabis as a potential treatment for some MHDs. As of June 2024, 24 states and the District of Columbia have recreational cannabis laws (RCLs) fully legalizing cannabis use for adults older than 21 years of age, and 30 states and the District of Columbia have medical cannabis laws (MCLs) allowing patients who received a diagnosis of a qualifying condition, including some MHDs, to access a medical cannabis card.^4^

Medical cannabis laws vary in the qualifying conditions that a patient must present with to gain access to a medical cannabis dispensary (the mechanism for access). It is not uncommon for states to include MHDs (eg, posttraumatic stress disorder and anxiety) in their list of qualifying conditions. However, the most common qualifying condition reported by medical card holders is chronic pain,^5^ the condition with the strongest evidence for cannabis treatment,^6^ and one often comorbid with MHDs.^7,8,9,10,11,12^ For individuals with such comorbid conditions, the therapeutic benefits associated with medical cannabis treatment may be much broader than just the treatment of pain, with the potential for unintended mental health benefits. This could lead to reductions in MHD-related drug dispensing, even without a medical cannabis card intended to treat MHDs.

In contrast to MCLs, RCLs cover the entire adult population, not just those with qualifying conditions. Thus, the numbers of potential users and medical conditions for which users could potentially benefit are much greater. Even if cannabis users are not intentionally consuming cannabis for medical purposes, the use itself may still affect mental health symptoms and subsequent psychotropic prescribing.

There is a sizable body of literature documenting the association between cannabis laws and prescribing of opioids.^13,14,15,16,17,18,19^ However, it is likely that these laws are also associated with psychotropic prescribing. Two prior studies^16,17^ found that MCLs were associated with reduced dispensing of drugs used to treat anxiety, depression, and sleep disorders among patients with Medicare and Medicaid. One study^17^ found that MCLs were associated with reductions in prescriptions for depression, anxiety, and sleep medications filled by Medicare enrollees, while the other study^16^ found no significant results for anxiety or sleep medications among Medicaid enrollees but did find reductions in dispensing of medications for depression. A more recent study^20^ found similar reductions for medications for anxiety, depression, and sleep among patients with Medicaid after the passage of RCLs. In addition, prior research indicates that the association between cannabis use and mental health symptoms is unclear, with some studies finding that cannabis use was positively associated with symptoms of anxiety, depression, and psychosis.^21,22,23,24^

We are unaware of studies investigating the association of MCLs or RCLs with psychotropic medication dispensing in the commercially insured population. We fill this gap by examining the associations of MCLs, RCLs, and cannabis dispensaries with dispensing for benzodiazepines (our primary outcome), as well as antidepressants, antipsychotics, barbiturates, and sleep medications in a large national claims database capturing data on privately insured populations.

## Methods

### Data and Measures

We extracted data from Optum’s deindentified Clinformatics Data Mart Database on commercially insured patients aged 18 to 64 years with prescription fills for each of the 5 medication classes: (1) benzodiazepines, (2) antidepressants, (3) antipsychotics, (4), barbiturates, and (5) sleep medications. Although this dataset includes information on a subset of Medicare Advantage patients, we excluded these enrollees because they represent a small amount of business for the plan sponsors and are not representative of Medicare Advantage enrollees nationwide. Our inclusion criteria were continuous insurance enrollment (≥6 months of continuous enrollment during the study period [January 1, 2007, to December 31, 2020]) and noncancer status (patients receiving a cancer diagnosis during the study period), as is standard in the literature.^25,26,27,28,29,30^ We followed the Strengthening the Reporting of Observational Studies in Epidemiology (STROBE) reporting guideline for cross-sectional studies. The University of Georgia institutional review board approved this study and waived the need for informed consent due to the deidentified nature of the data.

Prescription fills for the 5 drug classes were identified using American Hospital Formulary Service Pharmacologic–Therapeutic Classification System codes for benzodiazepines (282408), antidepressants (281604), antipsychotics (281608), barbiturates (282404), and sleep medications (282492).

### Outcome Variables

We constructed 3 outcome measures for each of the 5 drug classes. We calculated 1 extensive margin measure (the number of patients with prescriptions filled per 10 000 enrollees in each state and calendar quarter) and 2 intensive margin measures (the mean days’ supply per prescription fill and the mean number of prescription fills per patient each state and calendar quarter).

### Exposure Variables

Our primary exposure variables were 4 measures of medical or recreational cannabis access in each state quarter: (1) whether an MCL was in effect (ie, it was legal to possess cannabis with a medical certification, not just when the law was passed), (2) whether a state had open medical cannabis dispensaries, (3) whether an RCL was in effect (ie, it was legal for adults to possess cannabis for any reason, not just when the law was passed), and (4) whether a state had open recreational cannabis dispensaries. The information on dispensary openings came from a search of local news articles.

### Statistical Analysis

Statistical analysis was performed from September 2022 to November 2023. Recent literature has documented the limitations of a difference-in-differences framework in the presence of staggered adoption and heterogeneous treatment effects.^31,32^ There is reason to believe that the association of cannabis access with dispensing rates will change over time. This change could be due to the number of cannabis dispensaries changing, the number of patients substituting cannabis for pharmaceuticals changing, or the composition of patients in a state changing. Thus, we used a synthetic control strategy.^33,34^

In this method, a donor pool of never-treated states was used to construct a synthetic untreated outcome variable (eg, the counterfactual) estimate to pair with each treated state’s outcome series (see eFigures 1 and 2 in Supplement 1 for maps showing the states by their treatment group). The synthetic control procedure weights the donor (untreated) units’ outcome variable series to match the pretreatment outcome variable series for each treated unit. The aim is to construct a synthetic untreated series that matches as closely as possible to the observed series from the treated unit prior to the policy going into effect. Those same weights are used to construct a synthetic untreated outcome for the time periods when the treated unit is treated; this represents a counterfactual estimate of what would have happened to the outcome in the treated units had the policies not gone into effect. Thus, if (for example) medical dispensaries are associated with total dispensing rates, any deviation from the donor synthetic trend represents the treatment effect.^34,35^ This allows us to generate an overall treatment effect and a state-specific treatment effect (our case studies). We present the average treatment effect on the treated (ATT) states using a permutation inference.^36,37^ Thus, we will be able to assess which states are responsible for the overall association and whether there is any meaningful state heterogeneity. Finally, we performed a cross-fitting validation to account for overfitting (eg, a very sharp match of the synthetic control in the training data from the prepolicy period that biases the forecasts in the postperiod^34,38^). Our findings are robust to this form of misspecification. For a full description of this method, see the eAppendix in Supplement 1. *P* < .05 was considered statistically significant.

## Results

Prescription fills for MHDs were examined for 10 013 948 commercially insured patients. The benzodiazepine sample, our primary sample, included 3 848 721 individuals (mean [SD] age, 46.1 [11.4] years; 65.4% women and 34.6% men; 1.9% Asian individuals, 7.6% Black individuals, 8.4% Hispanic individuals, and 82.0% White individuals; 18.3% aged 18-34 years, 53.7% aged 35-54 years, and 28.1% aged 55-64 years). Baseline rates of drug dispensing are presented in eTable 1 in Supplement 1. Demographic characteristics by drug class and treatment status can be found in eTables 2 to 26 in Supplement 1. Selected results from our analysis of benzodiazepine dispensing are presented in the Figure^33,34,36,37^ (full results in eFigure 3 in Supplement 1). The Figure^33,34,36,37^ represents the estimated treatment effects (separately for each state) and provides the overall ATT with indicators of statistical significance, baseline rates, and change that the ATT represents in the heading above each subgraph. In a synthetic control analysis, unlike with typical inference methods, statistical significance is indicated when the estimate falls outside the 95% CI of the placebo estimates (for which the treatment effect is zero by construction). We frame our findings in terms of the mean percentage change over the baseline (the ATT divided by the baseline rate, multiplied by 100). Tabular results for individual state ATTs and graphical mean treatment effects over time with distributions from all simulation runs corresponding to benzodiazepine dispensing are available in eTable 27 and eFigure 4 in Supplement 1. Cross-state heterogeneity is assessed by comparing the signs and magnitudes of statistically significant state ATT estimates. Models estimating the association of cannabis laws with the 4 other drug categories (antidepressants, antipsychotics, barbiturates, and sleep medications) produced less consistent and precisely estimated findings and thus will not be discussed in detail. Full results for those drug classes can be found in eFigures 5 to 12 in Supplement 1.

**Figure.**
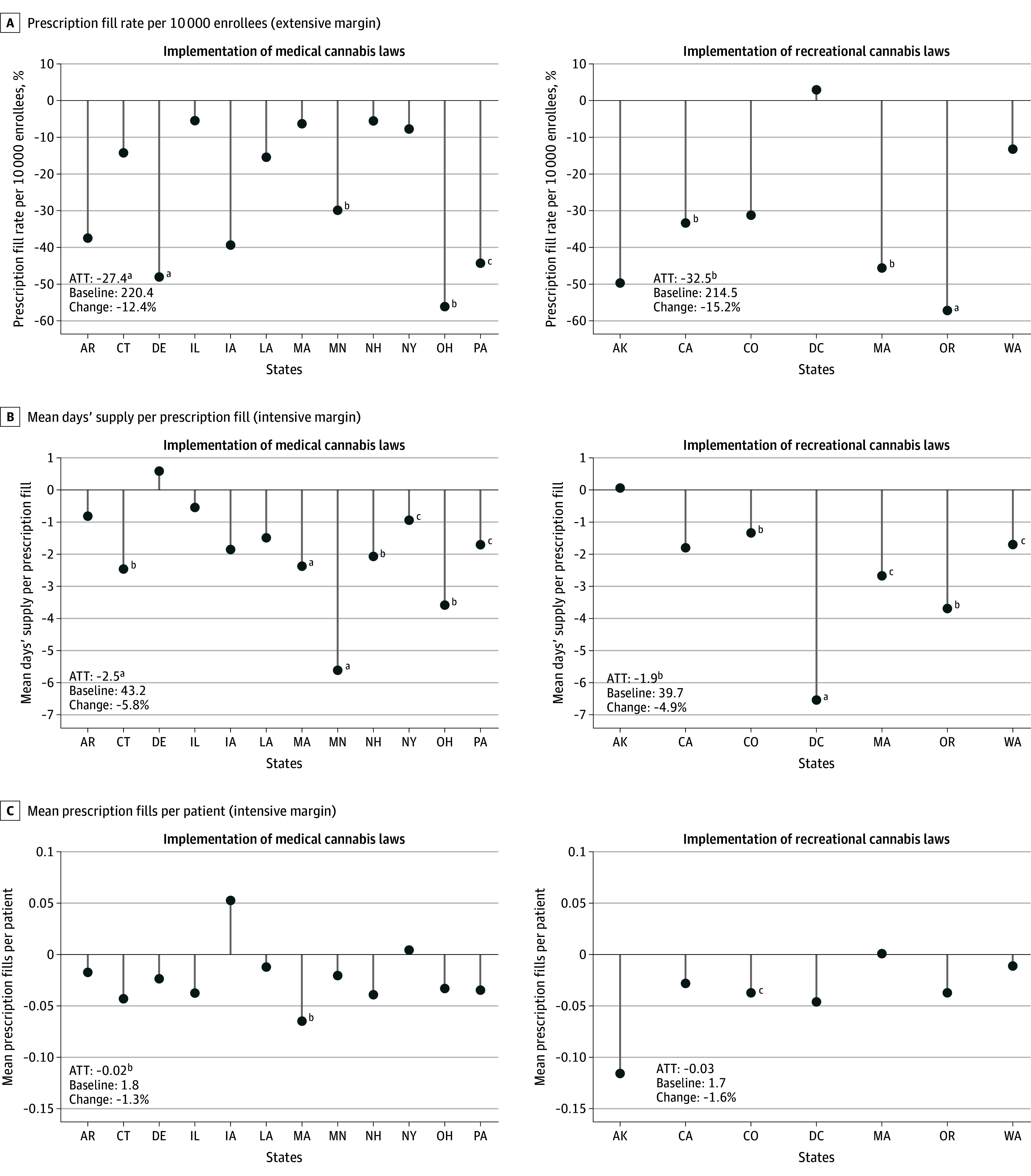
Benzodiazepine Prescription Fills: Case Study Mean Treatment Effects Calculations using information from Clinformatics aggregated at the state quarter level. Treatment effects comparing the actual and the synthetic series of outcome variables from individual case studies.^33,34^ The bottom left of each panel presents the average treatment effect on the treated (ATT) states and its comparison with the prepolicy baseline after the permutation inference.^36,37^ ^a^*P* < .01. ^b^*P* < .05. ^c^*P* < .10.

### Benzodiazepines

Both medical and recreational cannabis policies were consistently associated with reductions in benzodiazepine dispensing. Although we were unable to estimate a significant association for dispensary openings, we found that MCL implementation was associated with a 12.4% reduction in the prescription fill rate per 10 000 enrollees (the extensive margin) (ATT, −27.4; 95% CI, −14.7 to 12.0; *P* = .001) (Figure, A^33,34,36,37^; eTable 27 in Supplement 1, panel A). Recreational cannabis law implementation was associated with a 15.2% reduction in the fill rate (ATT, −32.5; 95% CI, −24.4 to 20.1; *P* = .02) (Figure, A^33,34,36,37^; eTable 27 in Supplement 1, panel B). Of the significant state case studies, there was no disagreement about the direction of the association. Each subexperiment produced estimates of a negative association of cannabis policies with the rate of benzodiazepine fills per 10 000 enrollees.

The estimates for the association of cannabis policies with our 2 measures of intensive margin benzodiazepine dispensing were smaller, although still significant. For the mean days’ supply per prescription fill, MCL implementation was associated with a 5.8% reduction (ATT, −2.5; 95% CI, −0.8 to 0.7; *P* < .001), and RCL implementation was associated with a 4.9% reduction (ATT, −1.9; 95% CI, −1.3 to 1.0; *P* = .01) (Figure, B^33,34,36,37^; eTable 27 in Supplement 1, panels A and B). Medical dispensaries were associated with a 3.9% reduction in mean days’ supply per prescription fill (ATT, −1.7; 95% CI, −0.8 to 0.6; *P* = .001), while recreational dispensaries showed a 6.2% reduction (ATT, −2.4; 95% CI, −1.0 to 0.9; *P* < .001) (eFigure 3, panel B, and eTable 27, panels A and B, in Supplement 1). Again, every state with significant results showed reductions in the mean days’ supply for benzodiazepine prescription fills.

Results for the mean number of benzodiazepine prescription fills per patient were less precisely and consistently estimated, although each ATT was found to be negative. Medical cannabis law implementation was associated with a 1.3% reduction (ATT, −0.02; 95% CI, −0.02 to 0.02; *P* = .04) (Figure, C^33,34,36,37^; eTable 27, panel A, in Supplement 1). The aggregate association of medical dispensaries with mean number of benzodiazepine prescription fills per patient was not significant at conventional levels, although several individual case studies were significant and negative (eFigure 3, panel C, and eTable 27, panel A, in Supplement 1). Finally, recreational dispensaries were associated with a 2.7% reduction in the mean number of benzodiazepine fills per patient (ATT, −0.04; 95% CI, −0.03 to 0.03; *P* = .01) (eFigure 3, panel C, and eTable 27, panel B, in Supplement 1). Only Colorado’s recreational dispensaries significantly contributed to this association.

### Other Drugs

In contrast to our results for benzodiazepines, our results for the other 4 classes of drugs were less precisely estimated (eFigures 5-12 in Supplement 1). We observed increases in antidepressant dispensing after cannabis policy enactment and dispensary openings. Medical cannabis law enactment was associated with a 3.8% increase in antidepressant fills per 10 000 enrollees (ATT, 27.2; 95% CI, −33.5 to 26.9; *P* = .048), while medical dispensaries were associated with an 8.8% increase (ATT, 50.7; 95% CI, −32.3 to 28.4; *P* = .004) (eFigure 5, panel A, and eTable 28, panel A, in Supplement 1), with minimal heterogeneity in the case studies. For our intensive margin measures, we estimated only 1 statistically significant result (a 2.7% increase in the mean days’ supply per fill [ATT, 2.3; 95% CI, −2.5 to 1.8; *P* = .02]) for medical dispensaries (eFigure 5, panel B, and eTable 28, panel A, in Supplement 1). Both RCL enactment and recreational dispensaries were associated with significant increases in the mean days’ supply of antidepressant fills, with no disagreements among the individual state case studies that contributed to the ATT (eFigure 5, panel B, and eTable 28, panel B, in Supplement 1).

Similar to antidepressants, we found evidence of an increase in dispensing of antipsychotic medications associated with cannabis legalization, although only for the intensive margins. After medical dispensary openings, the mean day’s supply per prescription fill increased by 2.6% (ATT, 2.0; 95% CI, −1.6 to 1.2; *P* = .008) with no disagreements among the significant case studies (eFigure 7, panel B, and eTable 29, panel A, in Supplement 1). The mean number of fills per patient increased after MCL implementation by 2.5% (ATT, 0.06; 95% CI, −0.04 to 0.05; *P* = .02) and after medical dispensary openings by 2.5% (ATT, 0.06; 95% CI, −0.04 to 0.04; *P* = .02) (eFigure 7, panel B, and eTable 29, panel C, in Supplement 1). There were no significant case studies that resulted in negative associations of medical dispensaries. Finally, RCL implementation was associated with a marginally significant increase in the number of fills per patient, but only Massachusetts and Washington showed individually significant results (eFigure 7, panel C, and eTable 29, panel B, in Supplement 1).

We also examined barbiturates and sleep medications. See eFigures 9-12 and eTable 30 and eTable 31 in Supplement 1 for detailed results. Results were not consistent and were often not significant at conventional levels. We found that RCLs were associated with reductions in the mean number of barbiturate prescription fills per patient, but those results were due almost entirely to California (eTable 30, panel B, in Supplement 1).

## Discussion

We found that cannabis laws and dispensaries were associated with significant decreases in the dispensing of benzodiazepines in a commercially insured population. Conversely, we found suggestive evidence that cannabis access was associated with increases in antidepressant and antipsychotic dispensing, although medical cannabis access had a more significant association at the extensive margin and recreational cannabis access had a more significant association at the intensive margin. Less-consistent results were found for barbiturates, and we were unable to estimate any significant associations for sleep medications.

Our findings are consistent with existing literature showing mixed results when examining the association between cannabis legalization and MHDs, particularly anxiety and depression. For example, Borbely et al^39^ found that cannabis laws have no discernible association with mental health outcomes broadly but do have differential associations by age group and type of cannabis law (MCL vs RCL). Specifically, MCLs were associated with improved self-reported mental health symptoms among older US adults, and RCLs were associated with worsened mental health symptoms among younger US adults (≤35 years). Another recent study showed that medical cannabis use was associated with an increased risk of emergency department visits for depressive disorders.^40^

These results have important implications for health outcomes. Medications used to treat anxiety, particularly benzodiazepines, are commonly misused and can be associated with serious medical conditions during withdrawal (such as delirium and seizures). In addition, benzodiazepine use can lead to harmful adverse effects, including respiratory depression, which can be fatal. Benzodiazepines, used in combination with opioids, accounted for 11 537 deaths in 2017, for 9711 deaths in 2019, and for 12 499 deaths in 2021.^41^ Such conditions do not occur with cannabis use, and there has never been a recorded death as a result of cannabis consumption, to our knowledge.^42^ Thus, if patients are, in fact, reducing their benzodiazepine use to manage their anxiety symptoms with cannabis, this may represent a safer treatment option overall.

Conversely, the positive association found between state cannabis laws and dispensing of antidepressants and antipsychotics is cause for concern, although perhaps unsurprising given the unsettled literature surrounding cannabis use and depression or psychosis. This finding indicates a need for additional investigations to explore how such access is associated with patient outcomes. This may be particularly prudent in the case of antipsychotics, as there is a growing body of evidence indicating that cannabis use may be associated with early onset of psychosis, acute psychosis, and worsening of psychotic disorders.^43,44,45^

### Limitations

This study has several limitations. First, we aggregated our data and therefore cannot make statements about how individuals respond (eg, the ecological fallacy). Second, we note that other policies were adopted throughout the study period. However, to the extent that the state-level adoption of other policies was associated with factors independent from those motivating MCL and RCL adoption, our estimated treatment effects will be valid. Third, we cannot firmly establish exactly when the change in access occurred, as one would expect substate heterogeneity in the degree to which such laws bind or the degree to which individuals can access dispensaries. Fourth, the large insurance system that is captured by Clinformatics has heterogeneous coverage across states. Fifth, this study is focused on how enrollees covered by commercial insurance products respond to cannabis access. Enrollees in exclusively managed care settings (Medicare Advantage and many state Medicaid programs) or low- or no-copayment plans for prescription drugs (state Medicaid plans) may have different responses to cannabis availability, as the gradient between subsidized prescription purchases and unsubsidized cannabis purchases will be larger than that for most commercially insured people.

## Conclusions

In this cross-sectional, synthetic control study of the US-based commercially insured population, we found consistent evidence that cannabis access was associated with reductions in dispensing of benzodiazepines and suggestive evidence that access was associated with increases in antidepressant and antipsychotic dispensing. Thus, access to cannabis may represent a meaningful shift in mental health treatment for this population. Our results have important implications for substance use and mental health–related outcomes. Specifically, the decreases in benzodiazepine dispensing suggest that there may be downstream aggregate decreases in the prevalence of adverse effects associated with benzodiazepine use. However, the increases in antidepressant and antipsychotic prescription fills may indicate a decrease in aggregate mental health outcomes. Overall, our results suggest that additional research is needed to assess whether changes in dispensing of MHDs are associated with differences in health care outcomes.

## Notes

Supplement 1. **eFigure 1.** Policy Variation in State Medical Cannabis **eFigure 2.** Policy Variation in State Recreational Cannabis **eTable 1.** Summary of Outcome Variables, by Treatment Status (2007 – 2020) **eTable 2.** Descriptive Statistics for Benzodiazepine Sample, All States **eTable 3.** Descriptive Statistics for Benzodiazepine Sample, MCL Legal **eTable 4.** Descriptive Statistics for Benzodiazepine Sample, MCL Dispensary **eTable 5.** Descriptive Statistics for Benzodiazepine Sample, RCL Legal **eTable 6.** Descriptive Statistics for Benzodiazepine Sample, RCL Dispensary **eTable 7.** Descriptive Statistics for Antidepressant Sample, All States **eTable 8.** Descriptive Statistics for Antidepressant Sample, MCL Legal **eTable 9.** Descriptive Statistics for Antidepressant Sample, MCL Dispensary **eTable 10.** Descriptive Statistics for Antidepressant Sample, RCL Legal **eTable 11.** Descriptive Statistics for Antidepressant Sample, RCL Dispensary **eTable 12.** Descriptive Statistics for Antipsychotic Sample, All States **eTable 13.** Descriptive Statistics for Antipsychotic Sample, MCL Legal **eTable 14.** Descriptive Statistics for Antipsychotic Sample, MCL Dispensary **eTable 15.** Descriptive Statistics for Antipsychotic Sample, RCL Legal **eTable 16.** Descriptive Statistics for Antipsychotic Sample, RCL Dispensary **eTable 17.** Descriptive Statistics for Barbiturate Sample, All States **eTable 18.** Descriptive Statistics for Barbiturate Sample, MCL Legal **eTable 19.** Descriptive Statistics for Barbiturate Sample, MCL Dispensary **eTable 20.** Descriptive Statistics for Barbiturate Sample, RCL Legal **eTable 21.** Descriptive Statistics for Barbiturate Sample, RCL Dispensary **eTable 22.** Descriptive Statistics for Sleep Medication Sample, All States **eTable 23.** Descriptive Statistics for Sleep Medication Sample, MCL Legal **eTable 24.** Descriptive Statistics for Sleep Medication Sample, MCL Dispensary **eTable 25.** Descriptive Statistics for Sleep Medication Sample, RCL Legal **eTable 26.** Descriptive Statistics for Sleep Medication Sample, RCL Dispensary **eAppendix.** Sample Construction and Methodology **eFigure 3.** Benzodiazepine Prescription Fills - Case Study Average Treatment Effects **eTable 27.** Average Treatment Effects Over Benzodiazepine Prescription Fills **eFigure 4.** Benzodiazepine Prescription Fills - Case Study Average Treatment Effects **eFigure 5.** Antidepressants’ Prescription Fills - Case Study Average Treatment Effects **eTable 28.** Average Treatment Effects Over Antidepressant Prescription Fills **eFigure 6.** Antidepressant Prescription Fills - Case Study Average Treatment Effects **eFigure 7.** Antipsychotic Prescription Fills - Case Study Average Treatment Effects **eTable 29.** Average Treatment Effects Over Antipsychotic Prescription Fills **eFigure 8.** Antipsychotic Prescription Fills - Case Study Average Treatment Effects **eFigure 9.** Barbiturate Prescription Fills - Case Study Average Treatment Effects **eTable 30.** Average Treatment Effects Over Barbiturate Prescription Fills **eFigure 10.** Barbiturate Prescription Fills - Case Study Average Treatment Effects **eFigure 11.** Sleep Medication Prescription Fills - Case Study Average Treatment Effects **eTable 31.** Average Treatment Effects Over Sleep Medication Prescription Fills **eFigure 12.** Sleep Medication Prescription Fills - Case Study Average Treatment Effects

Supplement 2.
Data Sharing Statement
