# Supplementary material for: Cannabis Laws and Utilization of Medications for the Treatment of Mental Health Disorders
Source: JAMA Netw Open. 2024 Sep 5;7(9):e2432021. doi: 10.1001/jamanetworkopen.2024.32021 (PMC11377998; doi:10.1001/jamanetworkopen.2024.32021)
Supplement: Supplement 1. — eFigure 1. Policy Variation in State Medical Cannabis eFigure 2. Policy Variation in State Recreational Cannabis eTable 1. Summary of Outcome Variables, by Treatment Status (2007 – 2020) eTable 2. Descriptive Statistics for Benzodiazepine Sample, All States eTable 3. Descriptive Statistics for Benzodiazepine Sample, MCL Legal eTable 4. Descriptive Statistics for Benzodiazepine Sample, MCL Dispensary eTable 5. Descriptive Statistics for Benzodiazepine Sample, RCL Legal eTable 6. Descriptive Statistics for Benzodiazepine Sample, RCL Dispensary eTable 7. Descriptive Statistics for Antidepressant Sample, All States eTable 8. Descriptive Statistics for Antidepressant Sample, MCL Legal eTable 9. Descriptive Statistics for Antidepressant Sample, MCL Dispensary eTable 10. Descriptive Statistics for Antidepressant Sample, RCL Legal eTable 11. Descriptive Statistics for Antidepressant Sample, RCL Dispensary eTable 12. Descriptive Statistics for Antipsychotic Sample, All States eTable 13. Descriptive Statistics for Antipsychotic Sample, MCL Legal eTable 14. Descriptive Statistics for Antipsychotic Sample, MCL Dispensary eTable 15. Descriptive Statistics for Antipsychotic Sample, RCL Legal eTable 16. Descriptive Statistics for Antipsychotic Sample, RCL Dispensary eTable 17. Descriptive Statistics for Barbiturate Sample, All States eTable 18. Descriptive Statistics for Barbiturate Sample, MCL Legal eTable 19. Descriptive Statistics for Barbiturate Sample, MCL Dispensary eTable 20. Descriptive Statistics for Barbiturate Sample, RCL Legal eTable 21. Descriptive Statistics for Barbiturate Sample, RCL Dispensary eTable 22. Descriptive Statistics for Sleep Medication Sample, All States eTable 23. Descriptive Statistics for Sleep Medication Sample, MCL Legal eTable 24. Descriptive Statistics for Sleep Medication Sample, MCL Dispensary eTable 25. Descriptive Statistics for Sleep Medication Sample, RCL Legal eTable 26. Descriptive Statistics for Sleep Medication Sample, RCL Dispensary e [file jamanetwopen-e2432021-s001.pdf]

Supplementary Online Content

Bradford AC, Lozano-Rojas F, Shone HB, Bradford WD, Abraham AJ. Cannabis laws and utilization of medications for the treatment of mental health disorders. *JAMA Netw Open.* 2024;7(9):e2432021.

doi:10.1001/jamanetworkopen.2024.32021

eFigure 1. Policy Variation in State Medical Cannabis
eFigure 2. Policy Variation in State Recreational Cannabis
eTable 1. Summary of Outcome Variables, by Treatment Status (2007 – 2020)
eTable 2. Descriptive Statistics for Benzodiazepine Sample, All States
eTable 3. Descriptive Statistics for Benzodiazepine Sample, MCL Legal
eTable 4. Descriptive Statistics for Benzodiazepine Sample, MCL Dispensary
eTable 5. Descriptive Statistics for Benzodiazepine Sample, RCL Legal
eTable 6. Descriptive Statistics for Benzodiazepine Sample, RCL Dispensary
eTable 7. Descriptive Statistics for Antidepressant Sample, All States
eTable 8. Descriptive Statistics for Antidepressant Sample, MCL Legal
eTable 9. Descriptive Statistics for Antidepressant Sample, MCL Dispensary
eTable 10. Descriptive Statistics for Antidepressant Sample, RCL Legal
eTable 11. Descriptive Statistics for Antidepressant Sample, RCL Dispensary
eTable 12. Descriptive Statistics for Antipsychotic Sample, All States
eTable 13. Descriptive Statistics for Antipsychotic Sample, MCL Legal
eTable 14. Descriptive Statistics for Antipsychotic Sample, MCL Dispensary
eTable 15. Descriptive Statistics for Antipsychotic Sample, RCL Legal
eTable 16. Descriptive Statistics for Antipsychotic Sample, RCL Dispensary
eTable 17. Descriptive Statistics for Barbiturate Sample, All States
eTable 18. Descriptive Statistics for Barbiturate Sample, MCL Legal
eTable 19. Descriptive Statistics for Barbiturate Sample, MCL Dispensary
eTable 20. Descriptive Statistics for Barbiturate Sample, RCL Legal
eTable 21. Descriptive Statistics for Barbiturate Sample, RCL Dispensary
eTable 22. Descriptive Statistics for Sleep Medication Sample, All States
eTable 23. Descriptive Statistics for Sleep Medication Sample, MCL Legal
eTable 24. Descriptive Statistics for Sleep Medication Sample, MCL Dispensary
eTable 25. Descriptive Statistics for Sleep Medication Sample, RCL Legal
eTable 26. Descriptive Statistics for Sleep Medication Sample, RCL Dispensary
eAppendix. Sample Construction and Methodology
eFigure 3. Benzodiazepine Prescription Fills - Case Study Average Treatment Effects
eTable 27. Average Treatment Effects Over Benzodiazepine Prescription Fills
eFigure 4. Benzodiazepine Prescription Fills - Case Study Average Treatment Effects
eFigure 5. Antidepressants' Prescription Fills - Case Study Average Treatment Effects
eTable 28. Average Treatment Effects Over Antidepressant Prescription Fills
eFigure 6. Antidepressant Prescription Fills - Case Study Average Treatment Effects
eFigure 7. Antipsychotic Prescription Fills - Case Study Average Treatment Effects
eTable 29. Average Treatment Effects Over Antipsychotic Prescription Fills

© 2024 Bradford AC et al. *JAMA Network Open.*

eFigure 8. Antipsychotic Prescription Fills - Case Study Average Treatment Effects
eFigure 9. Barbiturate Prescription Fills - Case Study Average Treatment Effects
eTable 30. Average Treatment Effects Over Barbiturate Prescription Fills
eFigure 10. Barbiturate Prescription Fills - Case Study Average Treatment Effects
eFigure 11. Sleep Medication Prescription Fills - Case Study Average Treatment Effects
eTable 31. Average Treatment Effects Over Sleep Medication Prescription Fills
eFigure 12. Sleep Medication Prescription Fills - Case Study Average Treatment Effects

This supplementary material has been provided by the authors to give readers additional information about their work.

eFigure 1. Policy Variation in State Medical Cannabis

MCL Legal

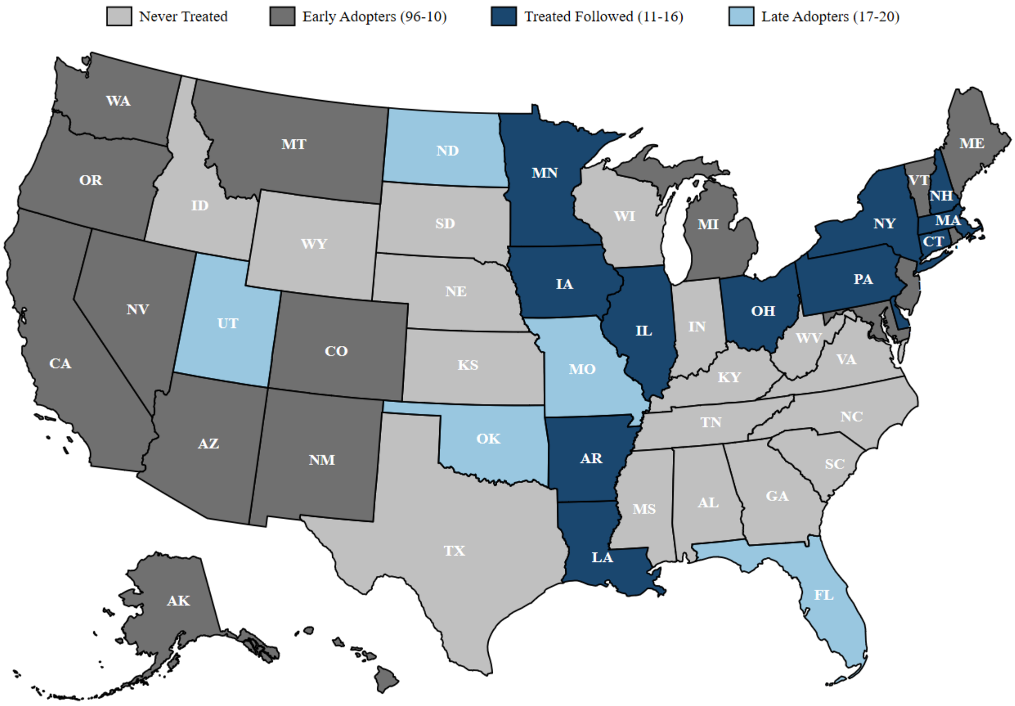

MCL Dispensary Open

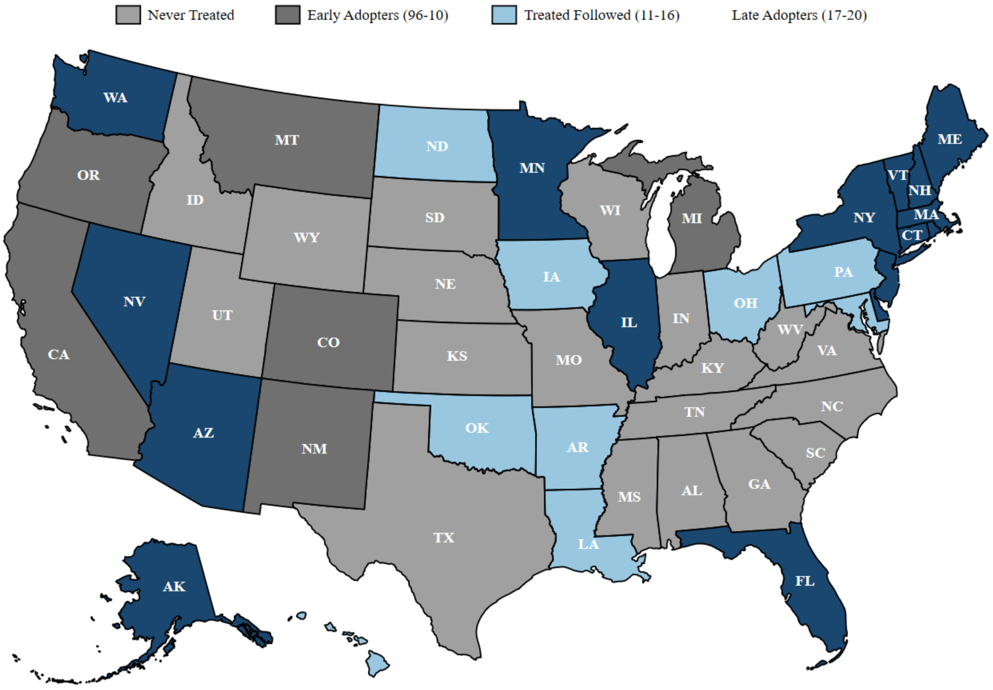

Note: The synthetic control analyses that we conducted included the "never treated" states and the "treated followed" states. Early adopters and late adopters were dropped from the sample. See eAppendix B for a full description of our statistical analysis. The MCL implementation analyses included 29 states (17 "never treated" and 12 "treated followed"). The MCL dispensary analyses included 36 states (19 "never treated" and 17 "treated followed").

© 2024 Bradford AC et al. *JAMA Network Open.*

eFigure 2. Policy Variation in State Recreational Cannabis

RCL Legal

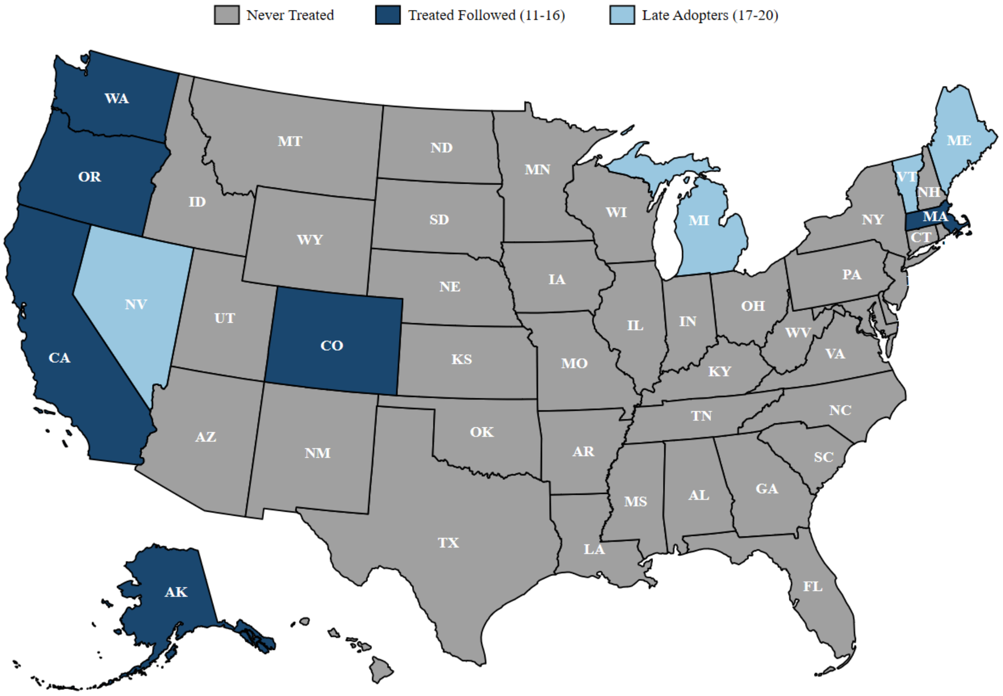

RCL Dispensary Open

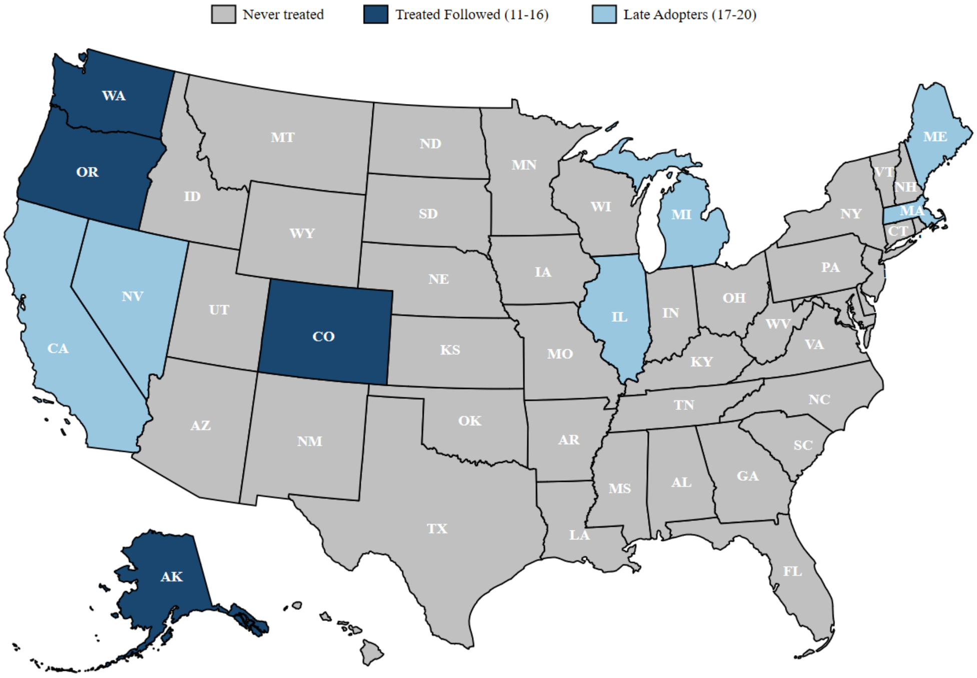

Note: The synthetic control analyses that we conducted included the “never treated” states and the “treated followed” states. Early adopters and late adopters were dropped from the sample. See eAppendix B for a full description of our statistical analysis. The RCL implementation analyses included 47 states (40 “never treated” and 7 “treated followed”). The RCL dispensary analyses included 45 states (41 “never treated” and 4 “treated followed”).

© 2024 Bradford AC et al. *JAMA Network Open.*

eTable 1. Summary of Outcome Variables, by Treatment Status (2007 – 2020)

	MCL Legal				MCL Dispensary Open				RCL Legal			RCL Dispensary Open		
**Medication - Outcome (Mean \ S.D.)**	**Never Treated**	**Treated Followed**	**Early Adopters**	**Late Adopters**	**Never Treated**	**Treated Followed**	**Early Adopters**	**Late Adopters**	**Never Treated**	**Treated Followed**	**Late Adopters**	**Never Treated**	**Treated Followed**	**Late Adopters**
Benzodiazepine - Prescription Rate	224.3	232.3	200.6	234.6	226.2	216.4	203.5	220.6	225.1	194.2	205.4	222.4	191.9	216.1
	(50.4)	(52.6)	(57.5)	(66.1)	(49.9)	(60.9)	(40.8)	(67.5)	(58.4)	(41.5)	(47.0)	(59.2)	(42.2)	(39.3)
Benzodiazepine - Avg. Days' Supply	45.7	43.4	40.6	44.9	45.7	41.3	40.3	44.4	44.5	37.5	42.1	44.2	36.0	43.0
	(4.9)	(4.3)	(5.1)	(5.9)	(4.8)	(5.2)	(4.5)	(5.0)	(4.8)	(4.4)	(5.6)	(5.2)	(4.3)	(3.7)
Benzodiazep. - Avg. Patient Prescrip.	1.86	1.78	1.74	1.86	1.86	1.73	1.74	1.84	1.82	1.69	1.76	1.81	1.71	1.76
	(0.103)	(0.120)	(0.113)	(0.111)	(0.099)	(0.117)	(0.077)	(0.123)	(0.116)	(0.109)	(0.113)	(0.125)	(0.109)	(0.076)
Antidepressant - Prescription Rate	743.0	734.0	601.7	750.7	750.4	634.3	631.9	732.0	719.7	591.2	623.6	716.4	618.3	595.8
	(111.2)	(152.4)	(168.2)	(153.7)	(112.0)	(162.9)	(117.1)	(205.5)	(157.6)	(122.6)	(149.8)	(158.8)	(115.6)	(141.4)
Antidepressant - Avg. Days' Supply	83.5	86.8	86.2	85.0	83.5	86.9	85.8	85.7	85.1	87.1	84.3	85.3	86.3	84.8
	(5.9)	(6.9)	(5.9)	(7.3)	(5.9)	(6.4)	(5.4)	(7.3)	(6.6)	(5.2)	(6.2)	(6.7)	(4.4)	(5.7)
Antidepress. - Avg. Patient Prescrip.	2.31	2.35	2.32	2.31	2.32	2.35	2.32	2.29	2.32	2.37	2.30	2.32	2.33	2.34
	(0.071)	(0.129)	(0.128)	(0.093)	(0.070)	(0.130)	(0.091)	(0.137)	(0.112)	(0.093)	(0.104)	(0.114)	(0.062)	(0.105)
Antipsychotic - Prescription Rate	46.5	49.6	41.3	46.8	47.5	44.8	42.5	44.8	47.0	42.1	36.7	46.5	41.1	42.3
	(11.0)	(11.0)	(11.2)	(13.7)	(11.5)	(12.3)	(8.7)	(12.5)	(11.8)	(9.0)	(10.9)	(12.2)	(9.6)	(8.8)
Antipsychotic - Avg. Days' Supply	74.9	78.2	77.0	74.7	74.9	77.7	78.0	75.9	76.1	77.8	76.1	76.1	77.3	77.5
	(4.9)	(5.1)	(7.7)	(4.4)	(4.8)	(7.3)	(4.2)	(6.4)	(5.5)	(5.4)	(10.8)	(6.3)	(6.1)	(5.0)
Antipsychot. - Avg. Patient Prescrip.	2.30	2.41	2.33	2.29	2.30	2.38	2.34	2.32	2.33	2.35	2.33	2.33	2.30	2.37
	(0.134)	(0.157)	(0.232)	(0.127)	(0.130)	(0.231)	(0.147)	(0.173)	(0.162)	(0.179)	(0.325)	(0.184)	(0.196)	(0.154)
Barbiturate - Prescription Rate	1.5	1.6	1.2	1.3	1.5	1.3	1.1	1.6	1.5	1.1	1.0	1.5	1.0	1.3
	(0.9)	(0.7)	(0.9)	(0.5)	(0.8)	(0.8)	(0.5)	(1.0)	(0.8)	(0.7)	(0.9)	(0.9)	(0.7)	(0.6)
Barbiturate - Avg. Days' Supply	79.3	82.0	75.5	78.9	79.1	78.8	76.1	79.9	79.4	79.8	68.4	79.0	83.8	74.2
	(13.7)	(12.7)	(19.2)	(13.4)	(13.5)	(18.2)	(16.8)	(13.3)	(14.5)	(15.4)	(23.9)	(15.1)	(15.2)	(17.4)
Barbiturates - Avg. Patient Prescrip.	2.26	2.37	2.21	2.21	2.26	2.31	2.23	2.22	2.27	2.36	2.05	2.26	2.38	2.21
	(0.430)	(0.419)	(0.548)	(0.425)	(0.415)	(0.522)	(0.497)	(0.461)	(0.462)	(0.449)	(0.544)	(0.475)	(0.490)	(0.413)
Sleep Medication - Prescription Rate	185.6	164.4	156.4	187.3	186.7	150.1	163.1	182.9	176.8	157.2	138.1	176.0	175.5	134.0
	(36.5)	(47.3)	(36.9)	(42.4)	(36.0)	(31.3)	(39.6)	(54.1)	(41.7)	(40.1)	(27.9)	(41.6)	(39.1)	(27.2)
Sleep Medication - Avg. Days' Supply	57.4	56.5	54.8	57.3	57.4	55.1	54.8	57.2	57.0	53.1	55.1	56.8	53.9	54.6
	(4.3)	(4.2)	(4.3)	(3.6)	(4.3)	(4.4)	(3.6)	(4.0)	(4.1)	(4.1)	(4.3)	(4.3)	(4.5)	(3.4)
Sleep Medication - Avg. Patient Prescrip.	1.90	1.88	1.81	1.90	1.90	1.83	1.80	1.89	1.88	1.78	1.80	1.88	1.80	1.82
	(0.110)	(0.113)	(0.123)	(0.113)	(0.112)	(0.121)	(0.093)	(0.132)	(0.117)	(0.100)	(0.144)	(0.126)	(0.095)	(0.093)
No. States	17	12	17	5	19	17	6	9	40	7	4	41	4	6

Note: Mean and Standard Deviation of outcome variables across the different state groups used in our analyses as classified by the different policy levers. Medical Cannabis splits the sample across four groups: Never Treated, Treated Followed, Early and Late Adopters, according to the implementation status and the timing of their policy adoption in relationship to our timeframe. In the case of recreational cannabis there are no Early Adopters, as the first state implementing, Colorado, did so in 2014, which falls in our timeframe for policy adoption (2011-2016). For more detail about the selection of states see eAppendix B1.

© 2024 Bradford AC et al. *JAMA Network Open.*

eTable 2. Descriptive Statistics for Benzodiazepine Sample, All States

	1	
	All states	
	Mean	S.D.
Female	0.654	0.476
White	0.820	0.384
Black	0.076	0.265
Asian	0.019	0.137
Hispanic	0.084	0.278
Missing Race	0.070	0.255
Age 18-24	0.043	0.202
Age 25-34	0.140	0.347
Age 35-44	0.231	0.422
Age 45-54	0.306	0.461
Age 55-64	0.281	0.449
Observations	12972993	

Notes: This table presents summary statistics for the benzodiazepine patient sample, aggregated to the quarter level.

© 2024 Bradford AC et al. *JAMA Network Open.*

eTable 3. Descriptive Statistics for Benzodiazepine Sample, MCL Legal

	1		2		3		4	
	Never Treated		Treated Followed		Early Adopters		Late Adopters	
	Mean	S.D.	Mean	S.D.	Mean	S.D.	Mean	S.D.
Female	0.661	0.473	0.651	0.477	0.651	0.477	0.650	0.477
White	0.809	0.393	0.872	0.334	0.797	0.402	0.815	0.389
Black	0.109	0.311	0.068	0.252	0.044	0.204	0.066	0.248
Asian	0.014	0.119	0.016	0.124	0.033	0.179	0.013	0.113
Hispanic	0.068	0.252	0.045	0.207	0.126	0.332	0.107	0.309
Missing Race	0.069	0.253	0.067	0.250	0.074	0.262	0.070	0.255
Age 18-24	0.042	0.200	0.045	0.207	0.046	0.210	0.036	0.187
Age 25-34	0.137	0.344	0.148	0.355	0.143	0.350	0.130	0.336
Age 35-44	0.237	0.425	0.228	0.419	0.230	0.421	0.226	0.418
Age 45-54	0.308	0.462	0.299	0.458	0.304	0.460	0.313	0.464
Age 55-64	0.277	0.447	0.281	0.449	0.276	0.447	0.294	0.456
Observations	4640210		2786562		3173383		2355830	

Notes: This table presents summary statistics for the benzodiazepine patient sample, aggregated to the quarter level.

© 2024 Bradford AC et al. *JAMA Network Open.*

eTable 4. Descriptive Statistics for Benzodiazepine Sample, MCL Dispensary

	1<br>Never Treated		2<br>Treated Followed		3<br>Early Adopters		4<br>Late Adopters	
	Mean	S.D.	Mean	S.D.	Mean	S.D.	Mean	S.D.
Female	0.660	0.474	0.646	0.478	0.647	0.478	0.661	0.473
White	0.820	0.384	0.824	0.380	0.776	0.417	0.856	0.351
Black	0.103	0.303	0.050	0.218	0.031	0.174	0.101	0.301
Asian	0.014	0.118	0.019	0.137	0.040	0.196	0.012	0.110
Hispanic	0.063	0.243	0.106	0.308	0.152	0.359	0.030	0.172
Missing Race	0.068	0.252	0.070	0.255	0.078	0.268	0.067	0.250
Age 18-24	0.042	0.200	0.043	0.203	0.046	0.209	0.040	0.196
Age 25-34	0.139	0.346	0.144	0.351	0.143	0.350	0.131	0.337
Age 35-44	0.237	0.425	0.226	0.418	0.233	0.423	0.225	0.417
Age 45-54	0.307	0.461	0.304	0.460	0.305	0.460	0.311	0.463
Age 55-64	0.276	0.447	0.283	0.451	0.274	0.446	0.294	0.456
Observations	5256912		3969842		1839465		1889766	

Notes: This table presents summary statistics for the benzodiazepine patient sample, aggregated to the quarter level.

© 2024 Bradford AC et al. *JAMA Network Open.*

eTable 5. Descriptive Statistics for Benzodiazepine Sample, RCL Legal

	1		2		3	
	Never Treated		Treated Followed		Late Adopters	
	Mean	S.D.	Mean	S.D.	Mean	S.D.
Female	0.656	0.475	0.647	0.478	0.647	0.478
White	0.825	0.380	0.787	0.410	0.869	0.338
Black	0.085	0.278	0.032	0.175	0.052	0.222
Asian	0.015	0.122	0.041	0.199	0.020	0.141
Hispanic	0.075	0.263	0.140	0.347	0.059	0.235
Missing Race	0.069	0.253	0.078	0.268	0.072	0.259
Age 18-24	0.042	0.200	0.046	0.210	0.048	0.214
Age 25-34	0.138	0.345	0.146	0.353	0.157	0.364
Age 35-44	0.231	0.421	0.234	0.423	0.238	0.426
Age 45-54	0.307	0.461	0.302	0.459	0.306	0.461
Age 55-64	0.283	0.450	0.271	0.445	0.251	0.433
Observations	10794909		1933030		245054	

Notes: This table presents summary statistics for the benzodiazepine patient sample, aggregated to the quarter level.

© 2024 Bradford AC et al. *JAMA Network Open.*

eTable 6. Descriptive Statistics for Benzodiazepine Sample, RCL Dispensary

	1		2		3	
	Never Treated		Treated Followed		Late Adopters	
	Mean	S.D.	Mean	S.D.	Mean	S.D.
Female	0.657	0.475	0.650	0.477	0.644	0.479
White	0.823	0.381	0.877	0.328	0.783	0.412
Black	0.087	0.282	0.026	0.158	0.037	0.188
Asian	0.015	0.121	0.021	0.143	0.040	0.197
Hispanic	0.075	0.263	0.077	0.266	0.139	0.346
Missing Race	0.068	0.252	0.067	0.249	0.080	0.271
Age 18-24	0.041	0.199	0.050	0.217	0.047	0.211
Age 25-34	0.138	0.344	0.158	0.365	0.144	0.351
Age 35-44	0.231	0.421	0.246	0.430	0.228	0.420
Age 45-54	0.307	0.461	0.289	0.453	0.306	0.461
Age 55-64	0.283	0.451	0.258	0.438	0.275	0.446
Observations	10349998		689456		1933539	

Notes: This table presents summary statistics for the benzodiazepine patient sample, aggregated to the quarter level.

© 2024 Bradford AC et al. *JAMA Network Open.*

eTable 7. Descriptive Statistics for Antidepressant Sample, All States

	1	
	All states	
	Mean	S.D.
Female	0.692	0.462
White	0.842	0.364
Black	0.069	0.253
Asian	0.018	0.133
Hispanic	0.071	0.256
Missing Race	0.067	0.250
Age 18-24	0.078	0.268
Age 25-34	0.152	0.359
Age 35-44	0.235	0.424
Age 45-54	0.286	0.452
Age 55-64	0.250	0.433
Observations	38475605	

Notes: This table presents summary statistics for the antidepressant patient sample, aggregated to the quarter level.

© 2024 Bradford AC et al. *JAMA Network Open.*

eTable 8. Descriptive Statistics for Antidepressant Sample, MCL Legal

	1		2		3		4	
	Never Treated		Treated Followed		Early Adopters		Late Adopters	
	Mean	S.D.	Mean	S.D.	Mean	S.D.	Mean	S.D.
Female	0.701	0.458	0.683	0.465	0.686	0.464	0.694	0.461
White	0.826	0.379	0.889	0.314	0.819	0.385	0.842	0.364
Black	0.096	0.295	0.058	0.233	0.041	0.198	0.059	0.236
Asian	0.014	0.117	0.015	0.123	0.031	0.175	0.013	0.112
Hispanic	0.064	0.244	0.038	0.191	0.109	0.311	0.085	0.280
Missing Race	0.067	0.250	0.064	0.245	0.072	0.259	0.065	0.246
Age 18-24	0.074	0.262	0.084	0.277	0.079	0.269	0.075	0.263
Age 25-34	0.149	0.356	0.159	0.366	0.148	0.355	0.151	0.358
Age 35-44	0.240	0.427	0.231	0.422	0.231	0.422	0.231	0.422
Age 45-54	0.289	0.453	0.279	0.448	0.289	0.453	0.287	0.452
Age 55-64	0.248	0.432	0.247	0.432	0.253	0.435	0.256	0.436
Observations	14635228		9576207		8693543		5531047	

Notes: This table presents summary statistics for the antidepressant patient sample, aggregated to the quarter level.

© 2024 Bradford AC et al. *JAMA Network Open.*

eTable 9. Descriptive Statistics for Antidepressant Sample, MCL Dispensary

	1		2		3		4	
	Never Treated		Treated Followed		Early Adopters		Late Adopters	
	Mean	S.D.	Mean	S.D.	Mean	S.D.	Mean	S.D.
Female	0.700	0.458	0.682	0.466	0.686	0.464	0.692	0.462
White	0.837	0.370	0.851	0.356	0.802	0.399	0.875	0.330
Black	0.091	0.287	0.044	0.205	0.028	0.165	0.084	0.278
Asian	0.014	0.116	0.019	0.137	0.038	0.192	0.012	0.110
Hispanic	0.059	0.236	0.085	0.280	0.132	0.338	0.028	0.166
Missing Race	0.066	0.248	0.067	0.250	0.076	0.265	0.063	0.243
Age 18-24	0.076	0.265	0.081	0.272	0.077	0.266	0.079	0.269
Age 25-34	0.152	0.359	0.155	0.362	0.147	0.354	0.148	0.355
Age 35-44	0.240	0.427	0.227	0.419	0.235	0.424	0.232	0.422
Age 45-54	0.286	0.452	0.283	0.450	0.292	0.455	0.286	0.452
Age 55-64	0.246	0.431	0.254	0.435	0.250	0.433	0.255	0.436
Observations	16625010		10720573		4843242		6247200	

Notes: This table presents summary statistics for the antidepressant patient sample, aggregated to the quarter level.

© 2024 Bradford AC et al. *JAMA Network Open.*

eTable 10. Descriptive Statistics for Antidepressant Sample, RCL Legal

	1		2		3	
	Never Treated		Treated Followed		Late Adopters	
	Mean	S.D.	Mean	S.D.	Mean	S.D.
Female	0.694	0.461	0.681	0.466	0.686	0.464
White	0.846	0.361	0.813	0.390	0.890	0.312
Black	0.076	0.264	0.029	0.169	0.045	0.208
Asian	0.015	0.120	0.039	0.194	0.018	0.132
Hispanic	0.063	0.244	0.118	0.323	0.047	0.211
Missing Race	0.066	0.248	0.076	0.265	0.073	0.260
Age 18-24	0.078	0.267	0.077	0.267	0.086	0.280
Age 25-34	0.151	0.358	0.152	0.359	0.167	0.373
Age 35-44	0.235	0.424	0.236	0.425	0.235	0.424
Age 45-54	0.286	0.452	0.289	0.453	0.281	0.449
Age 55-64	0.251	0.434	0.246	0.431	0.232	0.422
Observations	32563731		5292299		619575	

Notes: This table presents summary statistics for the antidepressant patient sample, aggregated to the quarter level.

© 2024 Bradford AC et al. *JAMA Network Open.*

eTable 11. Descriptive Statistics for Antidepressant Sample, RCL Dispensary

	1		2		3	
	Never Treated		Treated Followed		Late Adopters	
	Mean	S.D.	Mean	S.D.	Mean	S.D.
Female	0.695	0.461	0.688	0.463	0.677	0.468
White	0.845	0.362	0.884	0.320	0.806	0.395
Black	0.077	0.267	0.025	0.156	0.033	0.180
Asian	0.015	0.120	0.020	0.141	0.040	0.196
Hispanic	0.063	0.243	0.071	0.256	0.121	0.326
Missing Race	0.065	0.247	0.066	0.249	0.080	0.271
Age 18-24	0.077	0.267	0.077	0.266	0.082	0.275
Age 25-34	0.151	0.358	0.157	0.364	0.151	0.358
Age 35-44	0.235	0.424	0.245	0.430	0.229	0.420
Age 45-54	0.286	0.452	0.279	0.449	0.291	0.454
Age 55-64	0.251	0.434	0.242	0.428	0.246	0.431
Observations	31340185		2292314		4843106	

Notes: This table presents summary statistics for the antidepressant patient sample, aggregated to the quarter level.

© 2024 Bradford AC et al. *JAMA Network Open.*

eTable 12. Descriptive Statistics for Antipsychotic Sample, All States

	1	
	All states	
	Mean	S.D.
Female	0.597	0.491
White	0.813	0.390
Black	0.083	0.276
Asian	0.026	0.160
Hispanic	0.077	0.267
Missing Race	0.072	0.258
Age 18-24	0.165	0.371
Age 25-34	0.167	0.373
Age 35-44	0.216	0.412
Age 45-54	0.253	0.435
Age 55-64	0.200	0.400
Observations	2643790	

Notes: This table presents summary statistics for the antipsychotic patient sample, aggregated to the quarter level.

© 2024 Bradford AC et al. *JAMA Network Open.*

eTable 13. Descriptive Statistics for Antipsychotic Sample, MCL Legal

	1		2		3		4	
	Never Treated		Treated Followed		Early Adopters		Late Adopters	
	Mean	S.D.	Mean	S.D.	Mean	S.D.	Mean	S.D.
Female	0.614	0.487	0.580	0.494	0.588	0.492	0.596	0.491
White	0.800	0.400	0.861	0.346	0.790	0.408	0.812	0.391
Black	0.114	0.318	0.073	0.260	0.055	0.227	0.072	0.258
Asian	0.019	0.138	0.021	0.143	0.046	0.209	0.017	0.130
Hispanic	0.067	0.250	0.045	0.207	0.110	0.313	0.099	0.298
Missing Race	0.070	0.256	0.069	0.253	0.078	0.269	0.069	0.253
Age 18-24	0.160	0.366	0.167	0.373	0.173	0.378	0.160	0.367
Age 25-34	0.164	0.371	0.168	0.374	0.166	0.372	0.172	0.377
Age 35-44	0.225	0.418	0.209	0.406	0.212	0.408	0.214	0.410
Age 45-54	0.254	0.436	0.251	0.434	0.250	0.433	0.255	0.436
Age 55-64	0.197	0.397	0.206	0.404	0.199	0.399	0.198	0.399
Observations	961807		624607		672158		381412	

Notes: This table presents summary statistics for the antipsychotic patient sample, aggregated to the quarter level.

© 2024 Bradford AC et al. *JAMA Network Open.*

eTable 14. Descriptive Statistics for Antipsychotic Sample, MCL Dispensary

	1		2		3		4	
	Never Treated		Treated Followed		Early Adopters		Late Adopters	
	Mean	S.D.	Mean	S.D.	Mean	S.D.	Mean	S.D.
Female	0.612	0.487	0.578	0.494	0.588	0.492	0.599	0.490
White	0.812	0.391	0.821	0.384	0.778	0.416	0.838	0.368
Black	0.107	0.309	0.057	0.233	0.035	0.185	0.112	0.315
Asian	0.019	0.136	0.027	0.161	0.055	0.228	0.018	0.134
Hispanic	0.062	0.242	0.095	0.294	0.131	0.338	0.032	0.176
Missing Race	0.069	0.254	0.071	0.257	0.083	0.277	0.068	0.253
Age 18-24	0.161	0.368	0.168	0.374	0.172	0.377	0.162	0.368
Age 25-34	0.166	0.372	0.172	0.377	0.164	0.371	0.159	0.365
Age 35-44	0.225	0.417	0.207	0.405	0.212	0.409	0.214	0.410
Age 45-54	0.253	0.435	0.252	0.434	0.251	0.434	0.256	0.436
Age 55-64	0.195	0.396	0.200	0.400	0.201	0.401	0.210	0.408
Observations	1093226		769639		379639		397480	

Notes: This table presents summary statistics for the antipsychotic patient sample, aggregated to the quarter level.

© 2024 Bradford AC et al. *JAMA Network Open.*

eTable 15. Descriptive Statistics for Antipsychotic Sample, RCL Legal

	1		2		3	
	Never Treated		Treated Followed		Late Adopters	
	Mean	S.D.	Mean	S.D.	Mean	S.D.
Female	0.599	0.490	0.586	0.492	0.585	0.493
White	0.818	0.386	0.786	0.410	0.857	0.350
Black	0.092	0.289	0.037	0.189	0.062	0.241
Asian	0.021	0.143	0.055	0.229	0.027	0.162
Hispanic	0.070	0.254	0.122	0.327	0.055	0.228
Missing Race	0.070	0.255	0.082	0.275	0.086	0.280
Age 18-24	0.164	0.370	0.169	0.375	0.173	0.379
Age 25-34	0.166	0.372	0.166	0.373	0.181	0.385
Age 35-44	0.216	0.412	0.213	0.409	0.230	0.421
Age 45-54	0.253	0.435	0.251	0.434	0.235	0.424
Age 55-64	0.200	0.400	0.200	0.400	0.181	0.385
Observations	2190244		409654		43892	

Notes: This table presents summary statistics for the antipsychotic patient sample, aggregated to the quarter level.

© 2024 Bradford AC et al. *JAMA Network Open.*

eTable 16. Descriptive Statistics for Antipsychotic Sample, RCL Dispensary

	1		2		3	
	Never Treated		Treated Followed		Late Adopters	
	Mean	S.D.	Mean	S.D.	Mean	S.D.
Female	0.600	0.490	0.596	0.491	0.579	0.494
White	0.815	0.388	0.875	0.331	0.775	0.417
Black	0.094	0.292	0.029	0.167	0.043	0.202
Asian	0.021	0.142	0.024	0.153	0.058	0.233
Hispanic	0.069	0.254	0.072	0.259	0.124	0.330
Missing Race	0.069	0.254	0.066	0.248	0.089	0.285
Age 18-24	0.163	0.369	0.166	0.372	0.178	0.382
Age 25-34	0.166	0.372	0.165	0.371	0.169	0.375
Age 35-44	0.217	0.412	0.224	0.417	0.207	0.405
Age 45-54	0.254	0.435	0.249	0.432	0.249	0.432
Age 55-64	0.200	0.400	0.197	0.398	0.198	0.398
Observations	2101482		156622		385686	

Notes: This table presents summary statistics for the antipsychotic patient quarter, aggregated to the quarter level.

© 2024 Bradford AC et al. *JAMA Network Open.*

eTable 17. Descriptive Statistics for Barbiturate Sample, All States

	1	
	All states	
	Mean	S.D.
Female	0.555	0.497
White	0.791	0.407
Black	0.098	0.297
Asian	0.027	0.162
Hispanic	0.084	0.278
Missing Race	0.078	0.268
Age 18-24	0.063	0.243
Age 25-34	0.076	0.265
Age 35-44	0.145	0.352
Age 45-54	0.336	0.472
Age 55-64	0.379	0.485
Observations	78205	

Notes: This table presents summary statistics for the barbiturate patient sample, aggregated to the quarter level.

© 2024 Bradford AC et al. *JAMA Network Open.*

eTable 18. Descriptive Statistics for Barbiturate Sample, MCL Legal

	1 Never Treated		2 Treated Followed		3 Early Adopters		4 Late Adopters	
	Mean	S.D.	Mean	S.D.	Mean	S.D.	Mean	S.D.
Female	0.540	0.498	0.545	0.498	0.561	0.496	0.602	0.490
White	0.782	0.413	0.849	0.359	0.731	0.444	0.816	0.387
Black	0.137	0.344	0.089	0.285	0.063	0.244	0.072	0.259
Asian	0.017	0.131	0.022	0.147	0.053	0.224	0.015	0.121
Hispanic	0.063	0.244	0.040	0.196	0.153	0.360	0.097	0.296
Missing Race	0.074	0.261	0.078	0.268	0.087	0.282	0.071	0.257
Age 18-24	0.064	0.245	0.052	0.221	0.078	0.267	0.056	0.231
Age 25-34	0.070	0.254	0.069	0.254	0.080	0.272	0.100	0.300
Age 35-44	0.134	0.341	0.138	0.345	0.157	0.363	0.164	0.370
Age 45-54	0.333	0.471	0.353	0.478	0.332	0.471	0.323	0.467
Age 55-64	0.400	0.490	0.388	0.487	0.354	0.478	0.357	0.479
Observations	27843		20142		19133		10870	

Notes: This table presents summary statistics for the barbiturate patient sample, aggregated to the quarter level.

© 2024 Bradford AC et al. *JAMA Network Open.*

eTable 19. Descriptive Statistics for Barbiturate Sample, MCL Dispensary

	1<br>Never Treated		2<br>Treated Followed		3<br>Early Adopters		4<br>Late Adopters	
	Mean	S.D.	Mean	S.D.	Mean	S.D.	Mean	S.D.
Female	0.547	0.498	0.583	0.493	0.563	0.496	0.518	0.500
White	0.794	0.404	0.829	0.377	0.707	0.455	0.786	0.410
Black	0.130	0.336	0.046	0.210	0.036	0.187	0.161	0.368
Asian	0.017	0.127	0.031	0.174	0.055	0.228	0.022	0.147
Hispanic	0.060	0.237	0.094	0.292	0.201	0.401	0.031	0.174
Missing Race	0.074	0.262	0.081	0.273	0.094	0.292	0.068	0.252
Age 18-24	0.064	0.244	0.064	0.245	0.084	0.278	0.043	0.203
Age 25-34	0.073	0.261	0.080	0.271	0.086	0.281	0.070	0.256
Age 35-44	0.137	0.344	0.144	0.351	0.169	0.375	0.144	0.351
Age 45-54	0.333	0.471	0.342	0.474	0.331	0.470	0.340	0.474
Age 55-64	0.393	0.488	0.370	0.483	0.329	0.470	0.403	0.491
Observations	31258		23278		10502		12950	

Notes: This table presents summary statistics for the barbiturate patient sample, aggregated to the quarter level.

© 2024 Bradford AC et al. *JAMA Network Open.*

eTable 20. Descriptive Statistics for Barbiturate Sample, RCL Legal

	1		2		3	
	Never Treated		Treated Followed		Late Adopters	
	Mean	S.D.	Mean	S.D.	Mean	S.D.
Female	0.552	0.497	0.574	0.495	0.563	0.496
White	0.801	0.399	0.718	0.450	0.852	0.355
Black	0.109	0.312	0.035	0.185	0.039	0.195
Asian	0.022	0.147	0.056	0.231	0.029	0.168
Hispanic	0.067	0.250	0.190	0.392	0.079	0.270
Missing Race	0.074	0.262	0.100	0.300	0.075	0.263
Age 18-24	0.061	0.239	0.074	0.262	0.101	0.301
Age 25-34	0.074	0.262	0.089	0.284	0.087	0.282
Age 35-44	0.139	0.346	0.173	0.379	0.182	0.386
Age 45-54	0.335	0.472	0.350	0.477	0.273	0.446
Age 55-64	0.391	0.488	0.313	0.464	0.357	0.479
Observations	65689		11147		1369	

Notes: This table presents summary statistics for the barbiturate patient sample, aggregated to the quarter level.

© 2024 Bradford AC et al. *JAMA Network Open.*

eTable 21. Descriptive Statistics for Barbiturate Sample, RCL Dispensary

	1		2		3	
	Never Treated		Treated Followed		Late Adopters	
	Mean	S.D.	Mean	S.D.	Mean	S.D.
Female	0.550	0.497	0.632	0.482	0.563	0.496
White	0.797	0.402	0.864	0.343	0.742	0.438
Black	0.113	0.317	0.023	0.148	0.036	0.187
Asian	0.022	0.145	0.023	0.148	0.055	0.229
Hispanic	0.068	0.252	0.091	0.288	0.166	0.372
Missing Race	0.074	0.261	0.124	0.330	0.087	0.282
Age 18-24	0.060	0.238	0.049	0.217	0.083	0.276
Age 25-34	0.074	0.262	0.054	0.226	0.091	0.288
Age 35-44	0.140	0.347	0.119	0.324	0.178	0.382
Age 45-54	0.334	0.472	0.405	0.491	0.330	0.470
Age 55-64	0.392	0.488	0.372	0.484	0.317	0.465
Observations	62846		3040		12319	

Notes: This table presents summary statistics for the barbiturate patient sample, aggregated to the quarter level.

© 2024 Bradford AC et al. *JAMA Network Open.*

eTable 22. Descriptive Statistics for Sleep Medication Sample, All States

	1	
	All states	
	Mean	S.D.
Female	0.623	0.485
White	0.812	0.390
Black	0.083	0.275
Asian	0.025	0.156
Hispanic	0.080	0.272
Missing Race	0.068	0.252
Age 18-24	0.050	0.218
Age 25-34	0.125	0.331
Age 35-44	0.221	0.415
Age 45-54	0.312	0.463
Age 55-64	0.292	0.455
Observations	9908080	

Notes: This table presents summary statistics for the sleep medication patient sample, aggregated to the quarter level.

© 2024 Bradford AC et al. *JAMA Network Open.*

eTable 23. Descriptive Statistics for Sleep Medication Sample, MCL Legal

	1		2		3		4	
	Never Treated		Treated Followed		Early Adopters		Late Adopters	
	Mean	S.D.	Mean	S.D.	Mean	S.D.	Mean	S.D.
Female	0.636	0.481	0.619	0.486	0.610	0.488	0.620	0.485
White	0.797	0.403	0.859	0.348	0.796	0.403	0.816	0.388
Black	0.115	0.318	0.077	0.267	0.045	0.208	0.067	0.249
Asian	0.018	0.133	0.021	0.142	0.046	0.209	0.015	0.123
Hispanic	0.071	0.257	0.043	0.203	0.113	0.316	0.102	0.303
Missing Race	0.068	0.251	0.063	0.244	0.073	0.260	0.068	0.251
Age 18-24	0.050	0.218	0.056	0.230	0.047	0.211	0.048	0.214
Age 25-34	0.124	0.330	0.135	0.342	0.118	0.323	0.123	0.328
Age 35-44	0.228	0.419	0.216	0.411	0.217	0.412	0.217	0.412
Age 45-54	0.313	0.464	0.300	0.458	0.319	0.466	0.313	0.464
Age 55-64	0.285	0.451	0.293	0.455	0.300	0.458	0.299	0.458
Observations	3902502		2070593		2377204		1546055	

Notes: This table presents summary statistics for the sleep medication patient sample, aggregated to the quarter level.

© 2024 Bradford AC et al. *JAMA Network Open.*

eTable 24. Descriptive Statistics for Sleep Medication Sample, MCL Dispensary

	1		2		3		4	
	Never Treated		Treated Followed		Early Adopters		Late Adopters	
	Mean	S.D.	Mean	S.D.	Mean	S.D.	Mean	S.D.
Female	0.635	0.482	0.609	0.488	0.605	0.489	0.635	0.482
White	0.807	0.394	0.820	0.384	0.784	0.411	0.840	0.366
Black	0.108	0.311	0.053	0.224	0.029	0.168	0.113	0.316
Asian	0.018	0.131	0.025	0.156	0.056	0.230	0.016	0.125
Hispanic	0.067	0.249	0.102	0.302	0.131	0.337	0.031	0.174
Missing Race	0.067	0.250	0.069	0.253	0.076	0.264	0.062	0.242
Age 18-24	0.051	0.220	0.049	0.216	0.043	0.203	0.056	0.230
Age 25-34	0.127	0.333	0.127	0.333	0.114	0.318	0.126	0.332
Age 35-44	0.228	0.420	0.212	0.409	0.220	0.414	0.217	0.412
Age 45-54	0.311	0.463	0.310	0.462	0.325	0.468	0.305	0.460
Age 55-64	0.283	0.451	0.302	0.459	0.299	0.458	0.295	0.456
Observations	4340873		2704010		1404089		1447382	

Notes: This table presents summary statistics for the sleep medication patient sample, aggregated to the quarter level.

© 2024 Bradford AC et al. *JAMA Network Open.*

eTable 25. Descriptive Statistics for Sleep Medication Sample, RCL Legal

	1		2		3	
	Never Treated		Treated Followed		Late Adopters	
	Mean	S.D.	Mean	S.D.	Mean	S.D.
Female	0.628	0.483	0.600	0.490	0.612	0.487
White	0.815	0.389	0.795	0.404	0.859	0.348
Black	0.093	0.290	0.031	0.173	0.051	0.219
Asian	0.019	0.137	0.056	0.230	0.027	0.163
Hispanic	0.074	0.261	0.118	0.323	0.063	0.243
Missing Race	0.067	0.249	0.076	0.265	0.074	0.263
Age 18-24	0.051	0.220	0.043	0.203	0.056	0.229
Age 25-34	0.126	0.332	0.118	0.322	0.138	0.345
Age 35-44	0.221	0.415	0.221	0.415	0.220	0.414
Age 45-54	0.310	0.462	0.322	0.467	0.305	0.460
Age 55-64	0.292	0.455	0.296	0.456	0.281	0.450
Observations	8259780		1502085		146215	

Notes: This table presents summary statistics for the sleep medication patient sample, aggregated to the quarter level.

© 2024 Bradford AC et al. *JAMA Network Open.*

eTable 26. Descriptive Statistics for Sleep Medication Sample, RCL Dispensary

	1		2		3	
	Never Treated		Treated Followed		Late Adopters	
	Mean	S.D.	Mean	S.D.	Mean	S.D.
Female	0.629	0.483	0.611	0.488	0.597	0.490
White	0.813	0.390	0.881	0.324	0.773	0.419
Black	0.094	0.293	0.024	0.153	0.036	0.187
Asian	0.019	0.137	0.024	0.154	0.063	0.242
Hispanic	0.073	0.261	0.071	0.257	0.128	0.334
Missing Race	0.066	0.249	0.066	0.249	0.080	0.272
Age 18-24	0.051	0.220	0.045	0.208	0.046	0.210
Age 25-34	0.126	0.332	0.124	0.330	0.117	0.322
Age 35-44	0.221	0.415	0.228	0.419	0.215	0.411
Age 45-54	0.310	0.462	0.312	0.463	0.323	0.468
Age 55-64	0.292	0.454	0.291	0.454	0.298	0.458
Observations	8023421		630314		1254345	

Notes: This table presents summary statistics for the sleep medication patient sample, aggregated to the quarter level.

© 2024 Bradford AC et al. *JAMA Network Open.*

eAppendix. Sample Construction and Methodology

B1. Sample construction of State-Medication series

The analytic strategy we follow uses a series of synthetic control case-studies at the state-medication level on normalized series, following Abadie et al. (2010) (ADH). A separate synthetic control series is constructed for each treated state and policy considered (MCL/RCL Legal or MCL/RCL Dispensary Open). To do this, we construct balanced panels for each treated series and donor series. In this subsection, we establish the series that we follow and those that we use as donors. In the following subsection, we describe the statistical procedure.

We are interested in estimating the average treatment effect of each policy lever for those states that have enacted cannabis policies (Medical or Recreational Legal) and that have easy access (Dispensary Open). We define a window of analysis which guarantees that we can follow treated states for at least 16 quarters prior, the quarter of implementation, and for at least 16 quarters after, for a total of minimum 33 quarters. This allows us to evaluate the intermediate term effects of cannabis, as most of the literature has focused on the first two years. To illustrate this selection process, we give two examples. For instance, California, where medical cannabis has been legal since 1996, has its implementation date outside of our study timeframe. In the same fashion, Michigan enacted its recreational cannabis law in the last quarter of 2018, which would only let us follow their implementation for 8 quarters. As such, these state medication series are considered early adopters and late adopters, respectively, and not included in our analysis. In total, we follow 12 states for MCL Legal, 17 for MCL Dispensary Open, 7 for RCL Legal, and 4 for RCL Dispensary open.

The synthetic control series is constructed using a weighted average across the available pool of donor `never treated' state-medication series. We describe this weighted average in more detail in the next subsection. As of the end of the timeframe of our analyses, there were 17 states that had never been treated for MCL-Legal in 2020, 19 for MCL-Dispensary Open, 40 for RCL-Legal, and 43 for RCL-Dispensary Open. Figure eA-1 and Figure eA-2 showcase the states considered as Never Treated, Early adopters, Treated Followed, and Late Adopters for MCL and RCL, respectively, as of the end of 2020 when our timeframe ends.

So far, we have established the set of states that contribute to the treatment group (e.g. those that enacted the policies and that have enough calendar quarters in our timeframe), those in the donor group, and the never treated states. However, in each case we follow more than a medication series.

In the case of the treatment series, we explore their medication series individually. The results we present in Figures 1 thorough Figure 3 in the main article stem from the comparison of those individual state-medication series and the synthetic control constructed with the donors. In this article we are interested in medications commonly used to treat and manage a different range of mental health conditions. Hence, we follow benzodiazepines, antidepressants, antipsychotics, barbiturates, and sleep medications. The combination of one of this medication series and a treated state constitutes a case-study in our context.

For the analysis of an individual case-study, we pool a donor sample that includes the never treated states, pooling together all medications sampled. Accordingly, in the case of the donor series, we have all the never treated states multiplied by eight medication series. This would render a set of 320 donor series (8 medications × 40 never treated states) in the case of RCL Legal, for instance. We let this additional series to be part of the donor pool set as this considerably increases power and the likelihood of better matching the treated series of interest. We repeat this sample construction for each of the three

© 2024 Bradford AC et al. *JAMA Network Open.*

outcomes we consider in this article: rate of patients with prescription fills per 10,000 enrolled patients, the average days' supply per prescriptions fill, and the average number of prescription fills per patient in a given calendar quarter.

Hollingsworth and Wing (2020) suggest that, in addition to considering the series of interest, prescription fills of classes of drugs that are unlikely to be impacted by the pseudo version of the policy can contribute to the donor pool. This is the logic we follow here (Hollingsworth & Wing, 2020). The assumption remains the same: since the treatment does not affect the non-treated donor states on the outcomes of interest (mental health medications), it should also be true that the treatment does not affect prescriptions of these additional drug classes (pain management medications). As prescribing rates of each of the considered drug classes differ considerably, to facilitate comparison across case-studies, we normalize all series based on the pre-intervention period to exclusively exploit the variation on the medication series (Abadie 2021; Hollingsworth and Wing 2020).

While most synthetic control series estimation use only between 5 to 15 donor series to build a counterfactual (we explain this process below), we construct an empirical distribution of the estimated ATT parameters under conditions where we know the treatment effect is zero using the (untreated) donor pool sample for inference. In this case, we use all the series for which there is enough variation to construct a placebo synthetic control. Three states (AK, MT and VT) do not allow this to be the case as they have zero prescriptions of barbiturates in the first years of our timeframe. Following the example above of RCL Dispensary Open, instead of 320, we have 318 donor state-medication series, as we lose the barbiturates series for Montana and Vermont, and we cannot estimate a treatment effect for Alaska. Accordingly, while Alaska is reported in all other medications analyses, it is not reported in the analyses of barbiturates for RCL Dispensary Open. We further omit other state-medication series for inference based on the quality of their estimation, as we describe below, when we provide detail about the synthetic control method estimation we perform.

B.2 - Synthetic Control Method Estimation

Individual state synthetic control case-studies help overcome concerns about biases due to heterogeneity in the timing of policy adoption (early versus late adopters) and violations of parallel trends assumption in traditional difference-in-differences-based event studies. The proposed methodology also addresses concerns about the choices of clustering or functional forms on the estimation of parameters standard deviations, as it relies on placebo tests and randomization inference (Buchmueller et al. 2011; Hagemann 2019; Abadie 2021). We estimate each individual case-study, both from the treated and placebo series using the package *synth* in Stata 17 based in ADH 2010, 2015, and we augment it with current recommendations from the literature with regards to inference and goodness-of-fit assessment.

We are interested in the effect of state cannabis legalization and access (across four policy levers of MCL/RCL Legal or MCL/RCL Dispensary Open), in the states which have implemented them, on three measures of mental health medication prescribing (one extensive, two intensive) of five classes of medications (benzodiazepines, antidepressants, antipsychotics, barbiturates, and sleep-medications). Each medication series, Y_{it}, in state i and year t, can be defined as follows:

$$Y_{it} = Y_{it}^{N} + \alpha_{it} . D_t + \boldsymbol{\delta X_{it}} \ \ where\ D_t = \begin{cases} 1\ if\ i = 1\ and\ t \geq 0 \\ 0 \qquad\qquad otherwise \end{cases} \text{(Eq. 1)}$$

Where, in the context of potential outcomes notation, Y_{it}^{N} is the value of the prescription fill in the absence of treatment, and the treatment effect is α_{it} in state-medication and period observations with recreational cannabis policies (when $D_{it} = 1$).

© 2024 Bradford AC et al. *JAMA Network Open.*

Y_{it}^{N} is always observed for the never treated states. It is observed for treated states as well, but only in periods prior to the implementation of recreational cannabis policies. We estimate Y_{it}^{N} for treated states after treatment (separately for each policy lever), by estimating separate state-level case studies. In each case study, the treated state-medication is denoted by $i = 1$, as the remainder of the treated units are not considered jointly, such that in synthetic control estimation there is only a treated state-medication series. The counterfactual outcome of interest is denoted by $\widehat{Y_{it}^{N}}$, and we estimate this term using the ADH synthetic control method with the pool of donor units ($i \geq 2$). The synthetic control estimation procedure generates a set of weights $(w_2^*, \ldots, w_J^*)$, which are used to aggregate the contribution of all donor units to generate the "synthetic" control hat ($\widehat{Y_{it}^{N}}$). This synthetic series, $\widehat{Y_{it}^{N}}$, is constructed out of a selection of weights such that $\widehat{Y_{it}^{N}}$ approximates, as closely as possible, the treated series when $Y_{it} = Y_{it}^{N}$, in the pre-policy period ($t < 0$), by minimizing the root mean square prediction error (RMSPE[1]). Hence the synthetic series is the weighted average across donor series, using the set of weights $(w_2^*, \ldots, w_J^*)$ that minimize the RMSPE. The matrix of covariates $\boldsymbol{X_{it}}$ contains additional information including lags of the outcome variable. We include all available lags up to 20 quarters prior if available for each estimation of our synthetic control estimates.

The estimated synthetic control ($\widehat{Y_{it}^{N}}$), is then projected into the post-policy period and used as the counterfactual outcome against which the treated state's observed outcome is compared. The difference between the two is interpreted as the treatment effect of the policy in period t, represented as:

$$\widehat{\alpha_{1t}} = Y_{1t} - \widehat{Y_{1t}^{N}}, \text{ where } \widehat{Y_{1t}^{N}} = \sum_{i=2}^{J} w_i^* \cdot Y_{it} \quad (2)$$

The synthetic control method often provides very close matches in the pre-policy period to the state it is approximating, as can be seen in the smoke plots in the Appendix, as the pre-period the synthetic control of interest tends to be very close to zero and within the cloud of inference, which we will further describe in the next subsection.

We follow Hollingsworth and Wing (Hollingsworth & Wing, 2020) and average the differences between the actual series and the synthetic series for each period during the post-treatment time frame, up to four years post-policy, in order to recover DD-equivalent point estimates to summarize the treatment effects captured in the post-treatment period. This aggregation can be represented as:

$$\overline{\alpha_1} = \frac{1}{17} \cdot \sum_{t=0}^{T=16} \left(Y_{1t} - \widehat{Y_{1t}^{N}}\right) = \sum \left(\frac{\widehat{\alpha_{1t}}}{17}\right) \quad (3)$$

These are the treatment effects represented in Figure 1 through Figure three for the individual case studies and represented individually in each of the lollipop figures. The average treatment effects constitute average treatment effects on the treated, as stated on the main document. The average across all the treated state-medication series is also reported in the header of the text figures, and in the appendix tables for each of the five mental health medications of interest.

[1] The RMSPE is the average Euclidean distance between the treated series and the synthetic control during the pre-policy periods and measures how well the synthetic series replicates the treated series prior to the intervention. If it does have a good fit, it could be expected that it would have remained so in the absence of the policy in question.

© 2024 Bradford AC et al. *JAMA Network Open.*

B.3 - Synthetic Control Method Estimation

The ADH synthetic control model does not estimate both a parameter and standard error to all us to conduct statistical inference. Thus, we rely on placebo inference by comparing how large or small the effect on a given treated state is relative to the placebo treatment effects, calculated over the donor series from untreated states-medications (Abadie, 2021; Buchmueller et al., 2011; Hagemann, 2019). The key to understanding this approach is to remember that the "treatment effects" estimated from the placebo pools are drawn from a distribution that is know to be centered at zero – the true ATT equals zero for the placebo states. Inference is then based on determining whether our estimated ATT from the treated states falls outside the 95% confidence interval for the placebo estimated "treatment effects." Randomization based inference, as the one we implement in this article, has the advantage of not relying in additional assumptions about statistical distribution of the parameters of interest (Ding et al. 2016; MacKinnon & Webb, 2020).

We start by estimating each individual placebo treatment effect for each of the donor state-medication series. We want to consider donor series where the synthetic control can be estimated accurately. Hence, we trim 5% of placebo donor units with the highest RMSPE (Root Mean Square Prediction Error) in the pre-treatment period for each case study. We do this for each individual case study. Additionally, we assess fit by establishing if the actual series falls (in)outside of the bulk of the placebo distribution constructed from placebo synthetic controls for the pre-policy period. This is showcased in the smoke plot figures in the Appendix for all medications of interest, and we do not find any average treatment effect that would be challenged out of the lack of fit between treatment and synthetic control.

Our placebo test estimates the likelihood of attaining similarly large treatment effects if treatment were assigned to non-treated donor units. Specifically, we derive the p-value of the synthetic control treatment effects from the percentage of placebo treatment effects as large as the true treatment effects. The key assumption, similar to the DD assumptions, is that the true treatment effect only occurs for the actually treated states, at the timing of the treatment, with otherwise no systematic changes in outcomes in the placebo-treated units at the exact timing of the assigned placebo-treatment. The treatment effect can be considered statistically significant if the treatment effects fall outside the bulk (99%, 95%, or 90%) of the placebo treatment effects. This way of conducting hypothesis testing is referenced as Fisher Randomization Testing in the statistical literature (Ding et al. 2016).

So far, the inference methodology we have described allows us to estimate the treatment effect for a particular case-study exercise of a state-medication, but we are measuring the treatment effect across several treated states. To obtain the point estimate of the average treatment effect on the treated (ATT), we average across all treated case studies. For instance, we average across four states when following the effect of RCL Dispensary open, and across seven in RCL Legal. In the case of inference, we follow the procedure proposed by Cavallo et al. (2013), which draws samples of untreated series to approximate a placebo average treatment effect, one series for each treated unit. To each one of this randomly pulled placebo series, we assign a date of treatment considering the treatment dates of the actual treated states. We draw 5,000 combinations of the more than 150 (MCL Legal) and up to 387 donor series (RCL Dispensary Open) on the different treated states. The smoke plots presented in the Appendix report the series of treatment effects and the trace left by the placebo estimations used for inference. The period specific average treatment effect can be interpreted as significant if it falls outside the bulk of the distribution (99%, 95%, or 90%) of the placebo treatment effects, just as we described above for the case-study treatment effects.

With those placebo treatment effects, we have constructed a sharp null or an empirical distribution of a zero-treatment effect for each period after treatment. The smoke plots present the series of those differences, and we average the post-period treatment effect estimations to obtain the average placebo treatment effect in an analogous way as in Equation 3 above. In the tables, we report the standard deviation and confidence intervals based on this distribution.

eFigure 3. Benzodiazepine Prescription Fills - Case Study Average Treatment Effects

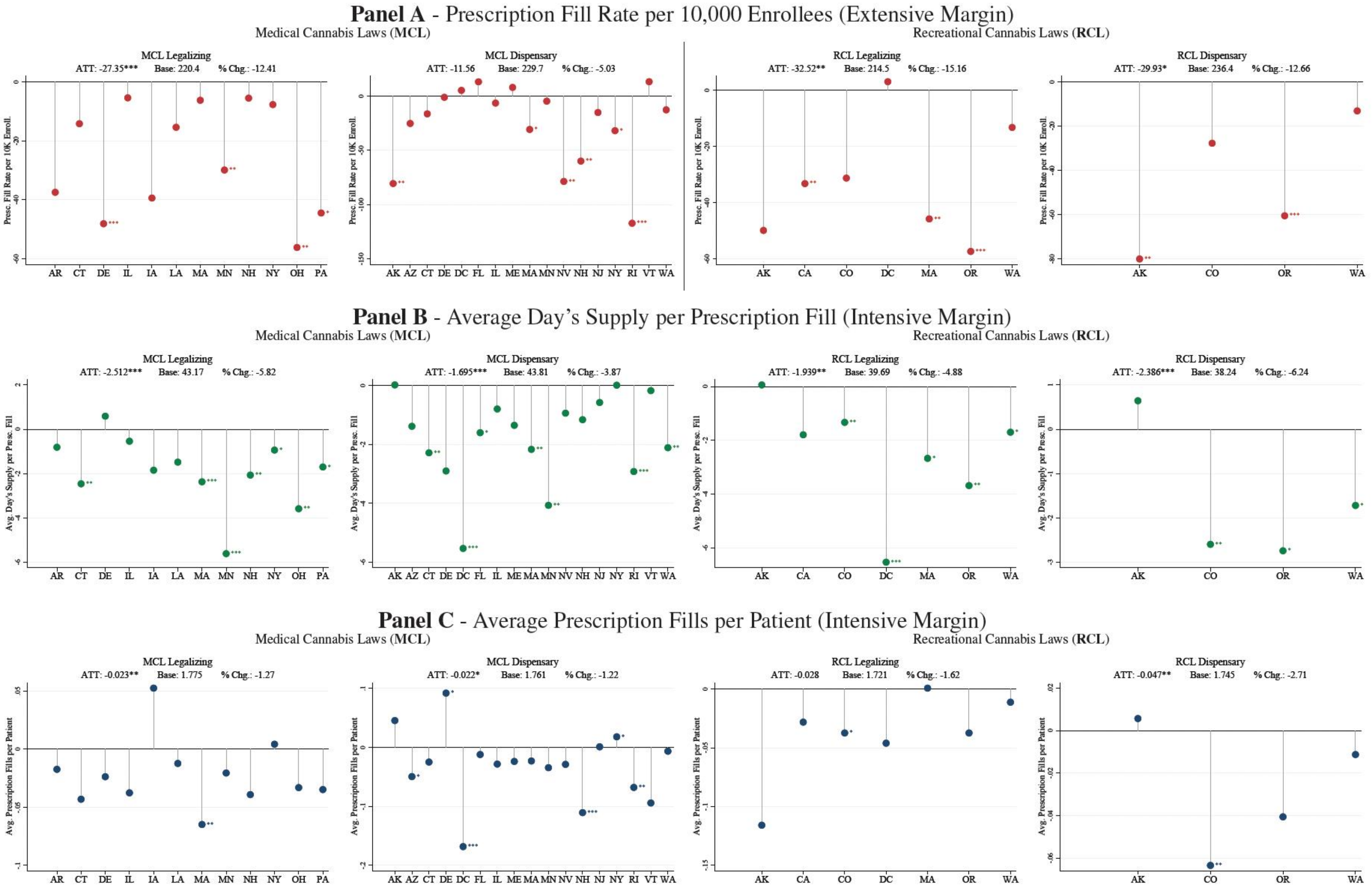

Notes: Authors' calculations using information from Clinformatics® aggregated at the state quarter level. Treatment effects comparing the actual and the synthetic series of outcome variables from individual case studies (Abadie et al. 2010; Abadie 2021). The top of each panel presents the average treatment effect on the treated states, and its comparison to the pre-policy baseline following the permutation inference (Cavallo et al. 2013; Hagemann 2019).

© 2024 Bradford AC et al. *JAMA Network Open.*

eTable 27. Average Treatment Effects Over Benzodiazepine Prescription Fills

PANEL A – MEDICAL CANNABIS LAWS

	ATT	Std. Dev.	95% C.I.	p-value	Baseline	%Chg.	+Cases	-Cases	Significant Case Studies
**Prescription Fill Rate per 10,000 Enrollees (Extensive Margin)**									
MCL Legal	-27.35***	8.14	[-14.66,12.04]	0.0010	220.4	-12.41	0	12	DE, MN, OH, PA
Dispensary Open	-11.56	13.94	[-24.48,21.61]	0.1886	229.7	-5.03	4	13	AK, MA, NV, NH, NY, RI
**Average Days' Supply per Prescription Fill (Intensive Margin)**									
MCL Legal	-2.512***	0.454	[-0.84,0.65]	0.0000	43.17	-5.82	1	11	CT, MA, MN, NH, NY, OH, PA
Dispensary Open	-1.695***	0.444	[-0.84,0.61]	0.0014	43.81	-3.87	2	15	CT, DC, FL, MA, MN, RI, WA
**Average Prescription Fills per Patient (Intensive Margin)**									
MCL Legal	-0.0226**	0.014	[-0.02,0.02]	0.0438	1.775	-1.27	2	10	MA
Dispensary Open	-0.0216*	0.013	[-0.02,0.02]	0.0514	1.761	-1.22	4	13	AZ, DE(+), DC, NH, NY(+), RI

PANEL B – RECREATIONAL CANNABIS LAWS

	ATT	Std. Dev.	95% C.I.	p-value	Baseline	%Chg.	+Cases	-Cases	Significant Case Studies
**Prescription Fill Rate per 10,000 Enrollees (Extensive Margin)**									
RCL Legal	-32.520**	13.92	[-24.43,20.05]	0.0172	214.5	-15.16	1	6	CA, MA, OR
Dispensary Open	-29.931*	24.85	[-48.25,31.28]	0.0978	236.4	-12.66	0	4	AK, OR
**Average Days' Supply per Prescription Fill (Intensive Margin)**									
RCL Legal	-1.939**	0.714	[-1.29,1.01]	0.0114	39.69	-4.88	1	6	CO, DC, MA, OR, WA
Dispensary Open	-2.386***	0.563	[-0.96,0.91]	0.0006	38.24	-6.24	1	6	CO, OR, WA
**Average Prescription Fills per Patient (Intensive Margin)**									
RCL Legal	-0.0279	0.026	[-0.04,0.04]	0.1486	1.721	-1.62	1	6	CO
Dispensary Open	-0.0473**	0.021	[-0.03,0.03]	0.0142	1.745	-2.71	1	3	CO

© 2024 Bradford AC et al. *JAMA Network Open.*

eFigure 4. Benzodiazepine Prescription Fills - Case Study Average Treatment Effects

Panel A - Prescription Fill Rate per 10,000 Enrollees (Extensive Margin)

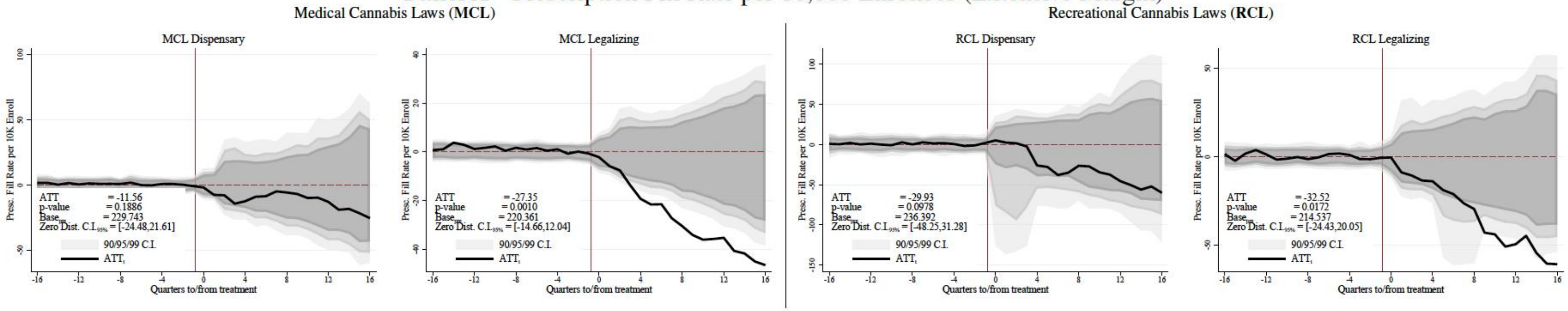

Panel B - Average Day's Supply per Prescription Fill (Intensive Margin)

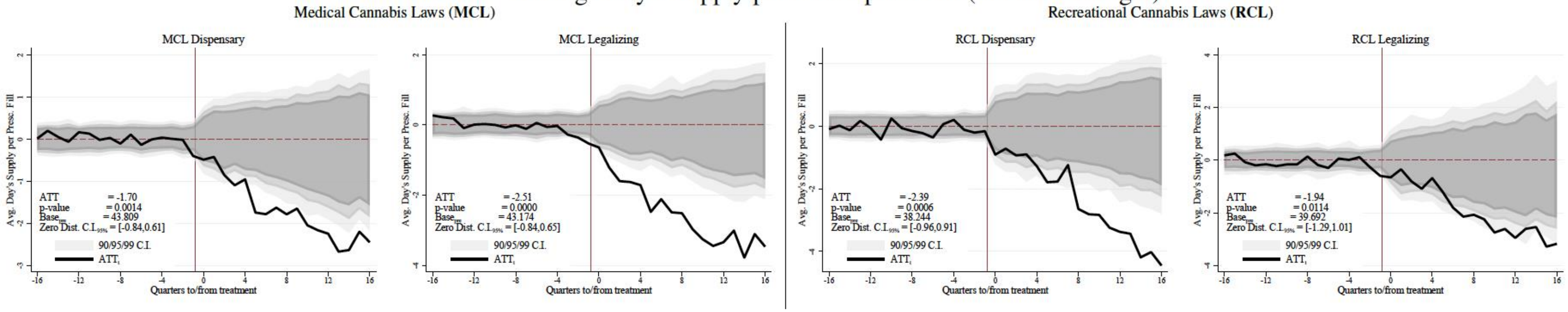

Panel C - Average Prescription Fills per Patient (Intensive Margin)

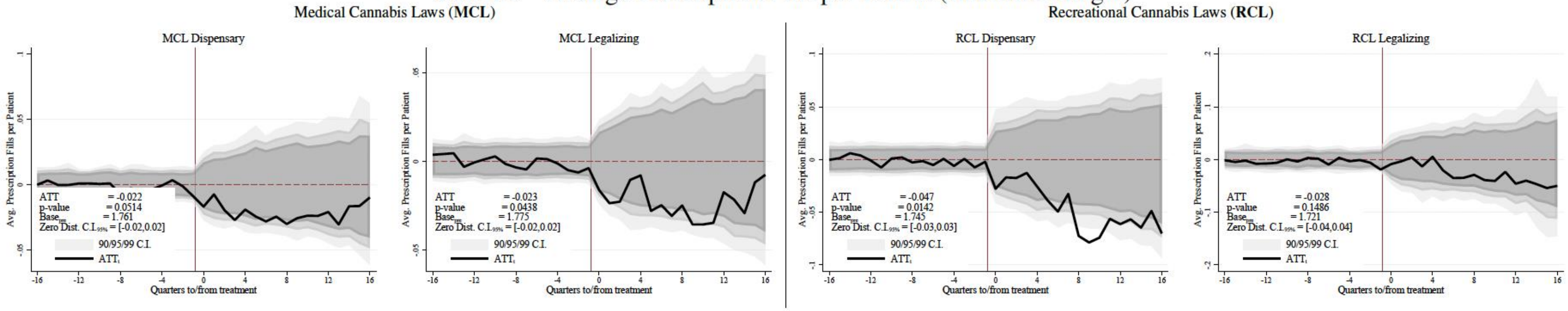

© 2024 Bradford AC et al. *JAMA Network Open.*

eFigure 5. Antidepressant Prescription Fills - Case Study Average Treatment Effects

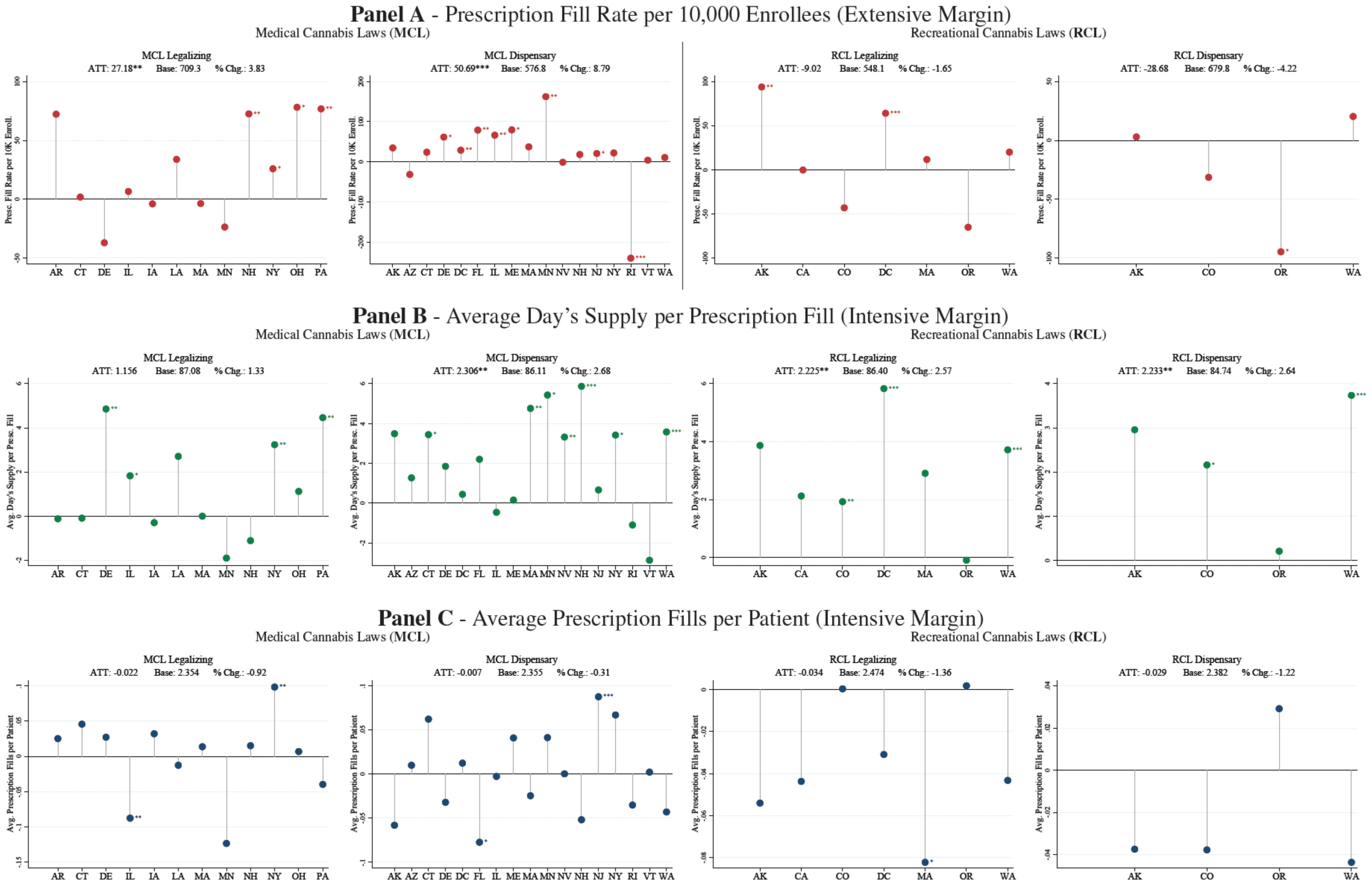

Notes: Authors' calculations using information from Clinformatics® aggregated at the state quarter level. Treatment effects comparing the actual and the synthetic series of outcome variables from individual case studies (Abadie et al. 2010; Abadie 2021). The top of each panel presents the average treatment effect on the treated states, and its comparison to the pre-policy baseline following the permutation inference (Cavallo et al. 2013; Hagemann 2019).

© 2024 Bradford AC et al. *JAMA Network Open.*

eTable 28. Average Treatment Effects Over Antidepressant Prescription Fills

PANEL A – MEDICAL CANNABIS LAWS

Prescription Fill Rate per 10,000 Enrollees (Extensive Margin)									
	**ATT**	**Std. Dev.**	**95% C.I.**	**p-value**	**Baseline**	**%Chg.**	**+Cases**	**-Cases**	**Significant Case Studies**
MCL Legal	27.181**	18.67	[-33.53,26.88]	0.0480	709.3	3.83	8	4	NH, NY, OH, PA
Dispensary Open	50.693***	18.40	[-32.25,28.39]	0.0044	576.8	8.79	14	3	DE, DC, FL, IL, ME, MN, NJ, RI(-)
**Average Days' Supply per Prescription Fill (Intensive Margin)**									
	**ATT**	**Std. Dev.**	**95% C.I.**	**p-value**	**Baseline**	**%Chg.**	**+Cases**	**-Cases**	**Significant Case Studies**
MCL Legal	1.156	1.26	[-2.18,1.91]	0.1586	87.08	1.33	6	6	DE, IL, NY, PA
Dispensary Open	2.306**	1.30	[-2.48,1.77]	0.0188	86.11	2.68	14	3	CT, MA, MN, NV, NH, NY, WA
**Average Prescription Fills per Patient (Intensive Margin)**									
	**ATT**	**Std. Dev.**	**95% C.I.**	**p-value**	**Baseline**	**%Chg.**	**+Cases**	**-Cases**	**Significant Case Studies**
MCL Legal	-0.0216	0.03	[-0.05,0.06]	0.2198	2.354	-0.92	8	4	IL, NY(+)
Dispensary Open	-0.0074	0.03	[-0.05,0.05]	0.4166	2.355	-0.31	9	8	FL, NJ(+)

PANEL B – RECREATIONAL CANNABIS LAWS

Prescription Fill Rate per 10,000 Enrollees (Extensive Margin)									
	**ATT**	**Std. Dev.**	**95% C.I.**	**p-value**	**Baseline**	**%Chg.**	**+Cases**	**-Cases**	**Significant Case Studies**
RCL Legal	-9.019	21.96	[-36.70,33.97]	0.3458	548.1	-1.65	4	3	AK(+), DC(+)
Dispensary Open	-28.676	36.68	[-72.71,45.28]	0.2006	679.8	-4.22	2	2	OR
**Average Days' Supply per Prescription Fill (Intensive Margin)**									
	**ATT**	**Std. Dev.**	**95% C.I.**	**p-value**	**Baseline**	**%Chg.**	**+Cases**	**-Cases**	**Significant Case Studies**
RCL Legal	2.225**	1.42	[-2.69,1.97]	0.0356	86.40	2.57	6	1	CO, DC, WA
Dispensary Open	2.233**	1.04	[-1.79,1.58]	0.0106	84.74	2.64	4	0	CO, WA
**Average Prescription Fills per Patient (Intensive Margin)**									
	**ATT**	**Std. Dev.**	**95% C.I.**	**p-value**	**Baseline**	**%Chg.**	**+Cases**	**-Cases**	**Significant Case Studies**
RCL Legal	-0.0337	0.04	[-0.07,0.06]	0.2168	2.474	-1.36	2	5	MA
Dispensary Open	-0.0290	0.04	[-0.07,0.07]	0.2292	2.382	-1.22	1	3	

© 2024 Bradford AC et al. *JAMA Network Open.*

eFigure 6. Antidepressant Prescription Fills - Case Study Average Treatment Effects

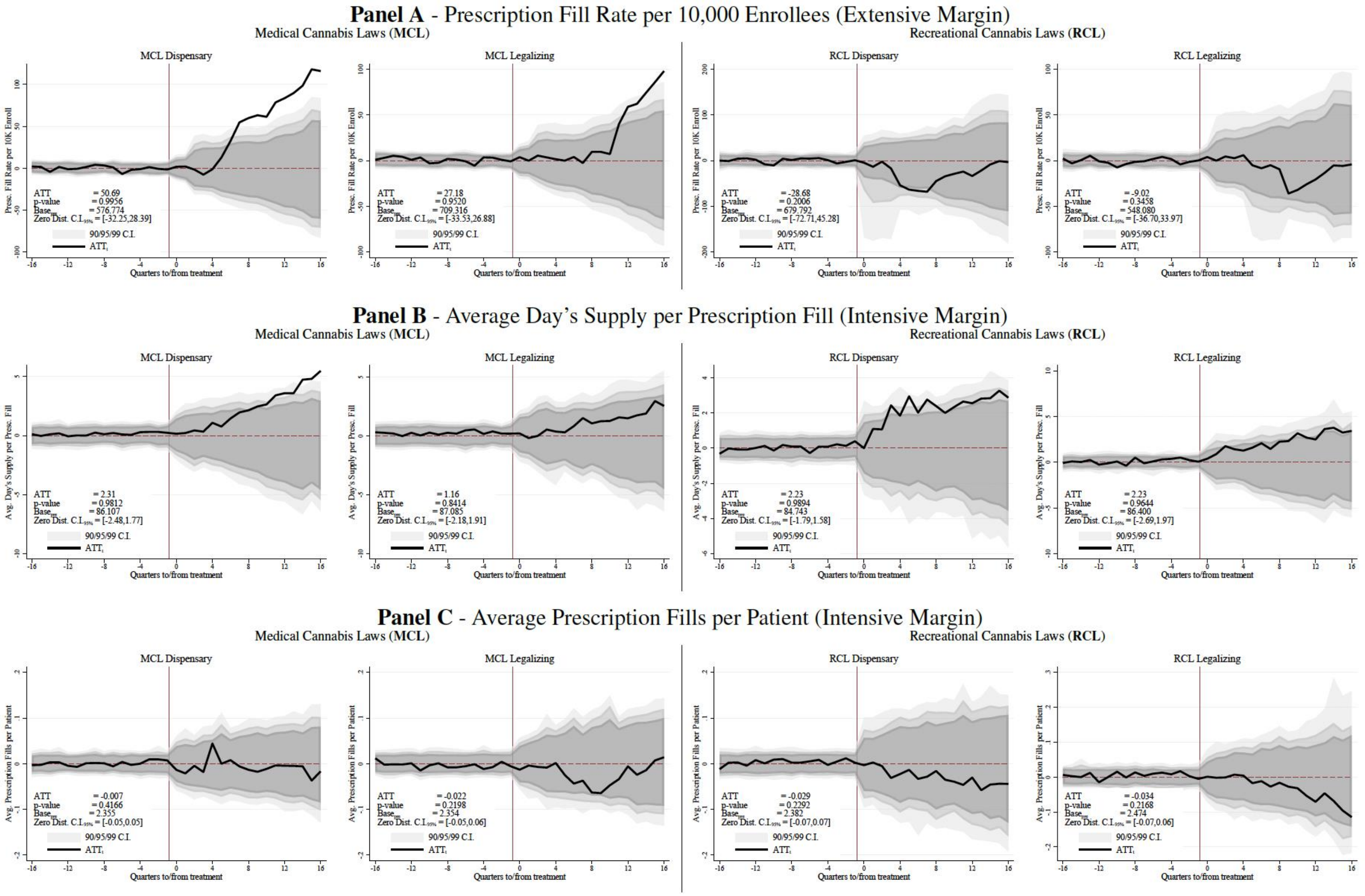

© 2024 Bradford AC et al. *JAMA Network Open.*

eFigure 7. Antipsychotic Prescription Fills - Case Study Average Treatment Effects

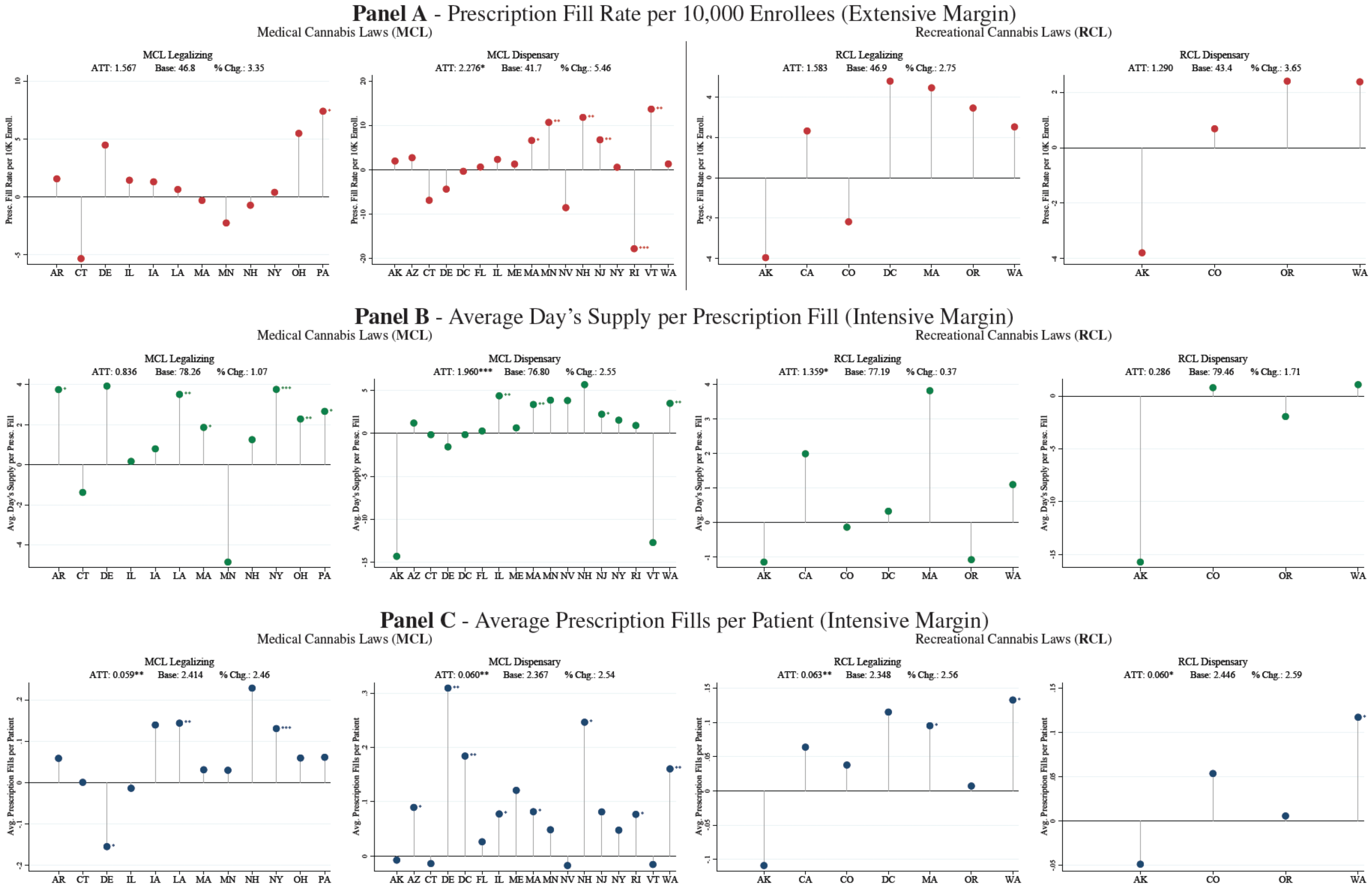

Notes: Authors' calculations using information from Clinformatics® aggregated at the state quarter level. Treatment effects comparing the actual and the synthetic series of outcome variables from individual case studies (Abadie et al. 2010; Abadie 2021). The top of each panel presents the average treatment effect on the treated states, and its comparison to the pre-policy baseline following the permutation inference (Cavallo et al. 2013; Hagemann 2019).

© 2024 Bradford AC et al. *JAMA Network Open.*

eTable 29. Average Treatment Effects Over Antipsychotic Prescription Fills

PANEL A – MEDICAL CANNABIS LAWS

	**ATT**	**Std. Dev.**	**95% C.I.**	**p-value**	**Baseline**	**%Chg.**	**+Cases**	**-Cases**	**Significant Case Studies**
**Prescription Fill Rate per 10,000 Enrollees (Extensive Margin)**									
MCL Legal	1.567	2.18	[-3.97,3.16]	0.1830	46.8	3.35	8	4	PA
Dispensary Open	2.276*	1.94	[-3.36,2.99]	0.0906	41.7	5.46	12	5	MA, MN, NH, NJ, RI(-), VT
**Average Days' Supply per Prescription Fill (Intensive Margin)**									
MCL Legal	0.836	0.94	[-1.67,1.46]	0.1620	78.26	1.07	10	2	AR, LA, MA, NY, OH, PA
Dispensary Open	1.960***	0.86	[-1.59,1.19]	0.0082	76.80	2.55	12	5	IL, MA, NJ, WA
**Average Prescription Fills per Patient (Intensive Margin)**									
MCL Legal	0.0593**	0.03	[-0.04,0.05]	0.0232	2.414	2.46	10	2	DE(-), LA, NY
Dispensary Open	0.0602**	0.03	[-0.04,0.04]	0.0166	2.367	2.54	13	4	AZ, DE, DC, IL, MA, NH, RI, WA

PANEL B – RECREATIONAL CANNABIS LAWS

	**ATT**	**Std. Dev.**	**95% C.I.**	**p-value**	**Baseline**	**%Chg.**	**+Cases**	**-Cases**	**Significant Case Studies**
**Prescription Fill Rate per 10,000 Enrollees (Extensive Margin)**									
RCL Legal	1.583	3.56	[-6.02,5.29]	0.2834	43.4	3.65	5	2	
Dispensary Open	1.290	5.63	[-11.42,6.94]	0.3786	46.9	2.75	3	1	
**Average Days' Supply per Prescription Fill (Intensive Margin)**									
RCL Legal	1.359*	1.15	[-2.11,1.62]	0.0798	79.46	1.71	4	3	
Dispensary Open	0.286	1.13	[-1.97,1.76]	0.3876	77.19	0.37	2	2	
**Average Prescription Fills per Patient (Intensive Margin)**									
RCL Legal	0.0634**	0.04	[-0.07,0.06]	0.0474	2.446	2.59	6	1	MA, WA
Dispensary Open	0.0601*	0.05	[-0.08,0.08]	0.0900	2.348	2.56	3	1	WA

© 2024 Bradford AC et al. *JAMA Network Open.*

eFigure 8. Antipsychotic Prescription Fills - Case Study Average Treatment Effects

Panel A - Prescription Fill Rate per 10,000 Enrollees (Extensive Margin)

Medical Cannabis Laws (**MCL**) | Recreational Cannabis Laws (**RCL**)

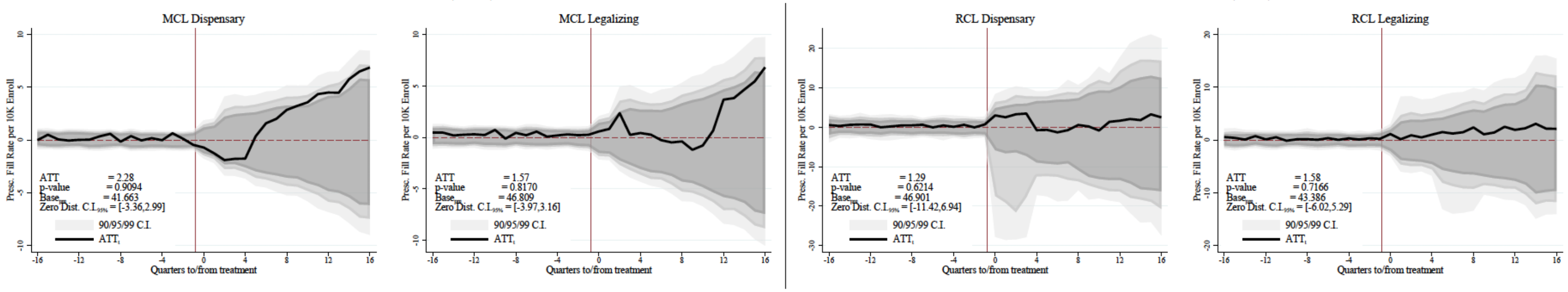

Panel B - Average Day's Supply per Prescription Fill (Intensive Margin)

Medical Cannabis Laws (**MCL**) | Recreational Cannabis Laws (**RCL**)

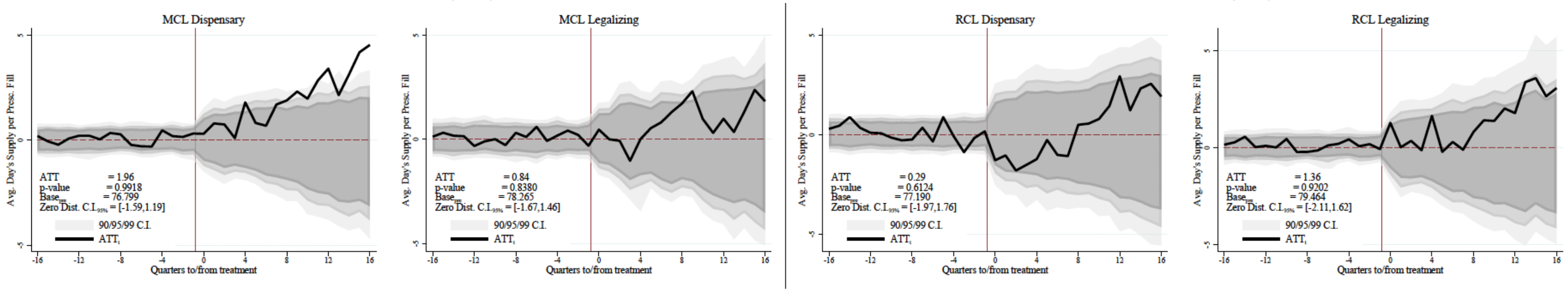

Panel C - Average Prescription Fills per Patient (Intensive Margin)

Medical Cannabis Laws (**MCL**) | Recreational Cannabis Laws (**RCL**)

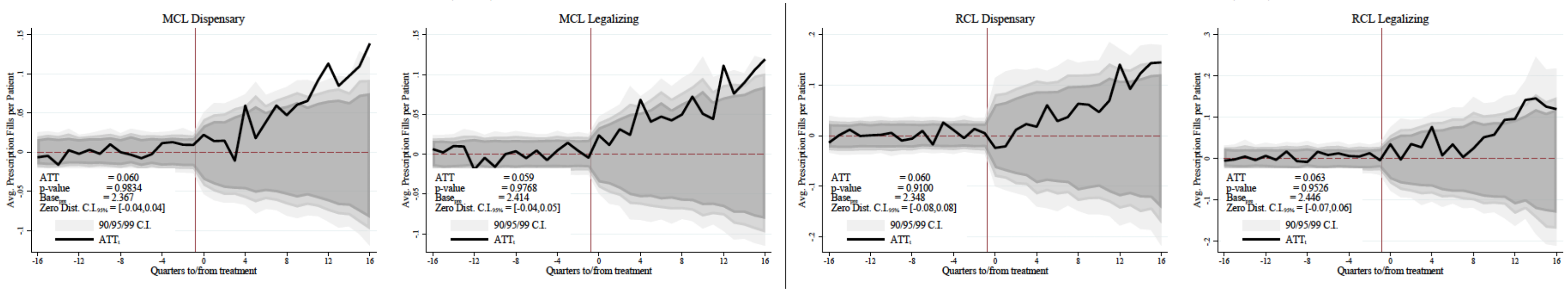

© 2024 Bradford AC et al. *JAMA Network Open.*

eFigure 9. Barbiturate Prescription Fills - Case Study Average Treatment Effects

Panel A - Prescription Fill Rate per 10,000 Enrollees (Extensive Margin)

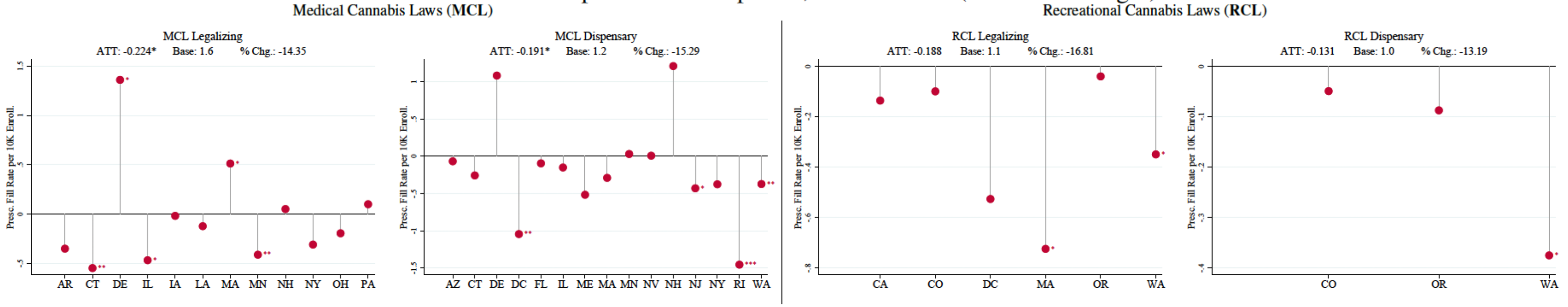

Panel B - Average Day's Supply per Prescription Fill (Intensive Margin)

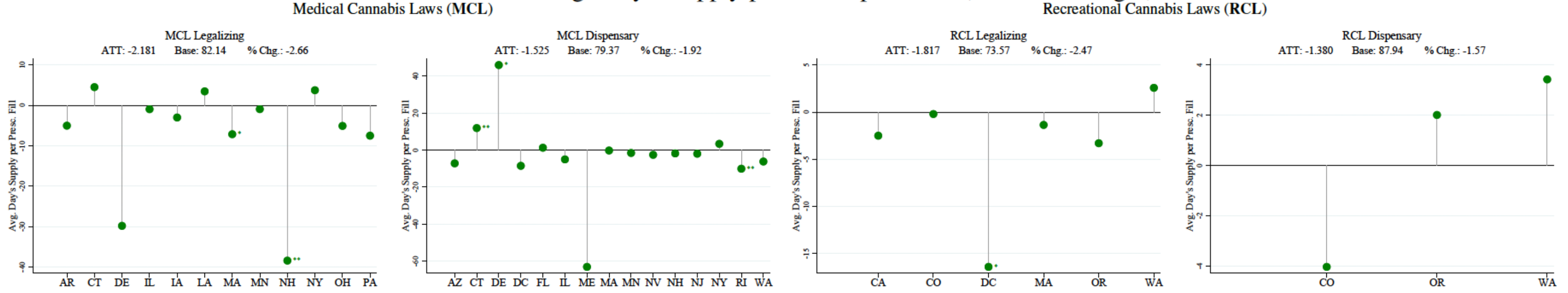

Panel C - Average Prescription Fills per Patient (Intensive Margin)

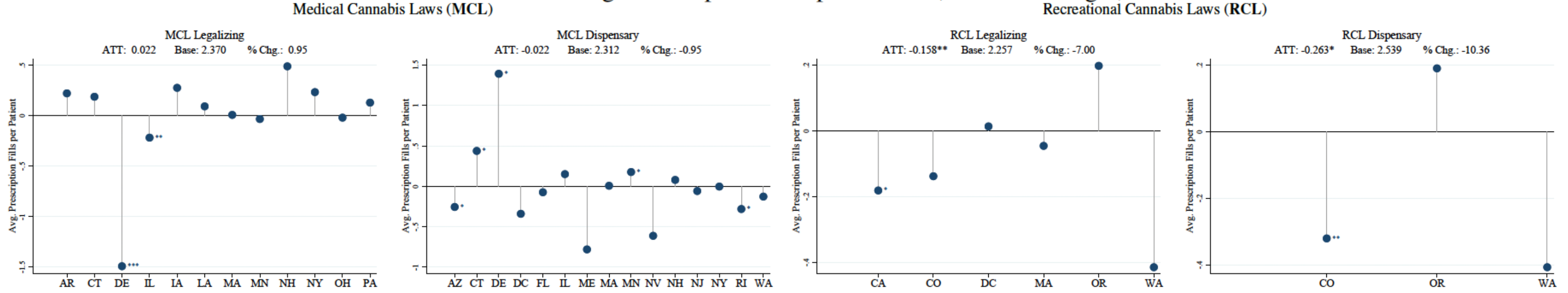

Notes: Authors' calculations using information from Clinformatics® aggregated at the state quarter level. Treatment effects comparing the actual and the synthetic series of outcome variables from individual case studies (Abadie et al. 2010; Abadie 2021). The top of each panel presents the average treatment effect on the treated states, and its comparison to the pre-policy baseline following the permutation inference (Cavallo et al. 2013; Hagemann 2019).

© 2024 Bradford AC et al. *JAMA Network Open.*

eTable 30. Average Treatment Effects Over Barbiturate Prescription Fills

PANEL A – MEDICAL CANNABIS LAWS

Prescription Fill Rate per 10,000 Enrollees (Extensive Margin)									
	**ATT**	**Std. Dev.**	**95% C.I.**	**p-value**	**Baseline**	**%Chg.**	**+Cases**	**-Cases**	**Significant Case Studies**
MCL Legal	-0.224*	0.14	[-0.25,0.20]	0.0732	1.6	-14.35	4	8	CT, DE(+), IL, MA(+), MN
Dispensary Open	-0.191*	0.14	[-0.24,0.22]	0.0972	1.2	-15.29	4	11	DC, NJ, RI, WA
**Average Days' Supply per Prescription Fill (Intensive Margin)**									
	**ATT**	**Std. Dev.**	**95% C.I.**	**p-value**	**Baseline**	**%Chg.**	**+Cases**	**-Cases**	**Significant Case Studies**
MCL Legal	-2.181	1.80	[-3.27,2.74]	0.1350	82.14	-2.66	3	9	MA, NH
Dispensary Open	-1.525	1.79	[-3.33,2.51]	0.2416	79.37	-1.92	4	11	CT(+), DE(+), RI
**Average Prescription Fills per Patient (Intensive Margin)**									
	**ATT**	**Std. Dev.**	**95% C.I.**	**p-value**	**Baseline**	**%Chg.**	**+Cases**	**-Cases**	**Significant Case Studies**
MCL Legal	0.022	0.06	[-0.09,0.10]	0.3612	2.370	0.95	8	4	DE(-), IL(-)
Dispensary Open	-0.022	0.06	[-0.09,0.09]	0.3452	2.312	-0.95	6	9	AZ, CT(+), DE(+), MN(+), RI

PANEL B – RECREATIONAL CANNABIS LAWS

Prescription Fill Rate per 10,000 Enrollees (Extensive Margin)									
	**ATT**	**Std. Dev.**	**95% C.I.**	**p-value**	**Baseline**	**%Chg.**	**+Cases**	**-Cases**	**Significant Case Studies**
RCL Legal	-0.188	0.18	[-0.30,0.27]	0.1272	1.1	-16.81	0	6	MA, WA
Dispensary Open	-0.131	0.15	[-0.29,0.21]	0.1892	1.0	-13.19	0	3	WA
**Average Days' Supply per Prescription Fill (Intensive Margin)**									
	**ATT**	**Std. Dev.**	**95% C.I.**	**p-value**	**Baseline**	**%Chg.**	**+Cases**	**-Cases**	**Significant Case Studies**
RCL Legal	-1.817	4.03	[-7.49,5.86]	0.3084	73.57	-2.47	1	5	DC
Dispensary Open	-1.380	5.18	[-8.53,8.14]	0.3626	87.94	-1.57	2	1	
**Average Prescription Fills per Patient (Intensive Margin)**									
	**ATT**	**Std. Dev.**	**95% C.I.**	**p-value**	**Baseline**	**%Chg.**	**+Cases**	**-Cases**	**Significant Case Studies**
RCL Legal	-0.158**	0.09	[-0.15,0.13]	0.0424	2.257	-7.00	2	4	CA
Dispensary Open	-0.263*	0.17	[-0.29,0.27]	0.0630	2.539	-10.36	1	2	CO

© 2024 Bradford AC et al. *JAMA Network Open.*

eFigure 10. Barbiturate Prescription Fills - Case Study Average Treatment Effects

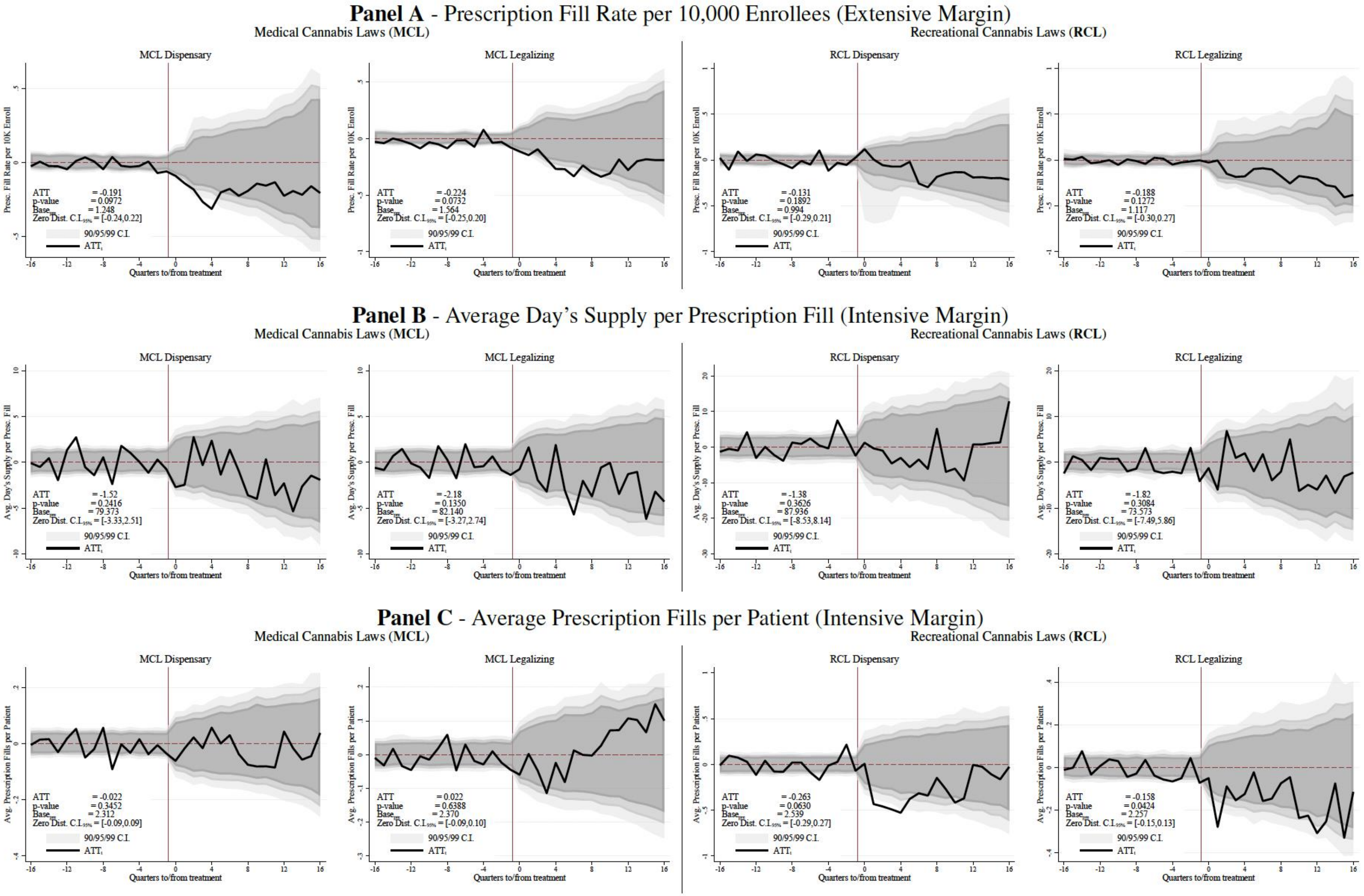

© 2024 Bradford AC et al. *JAMA Network Open.*

eFigure 11. Sleep Medication Prescription Fills - Case Study Average Treatment Effects

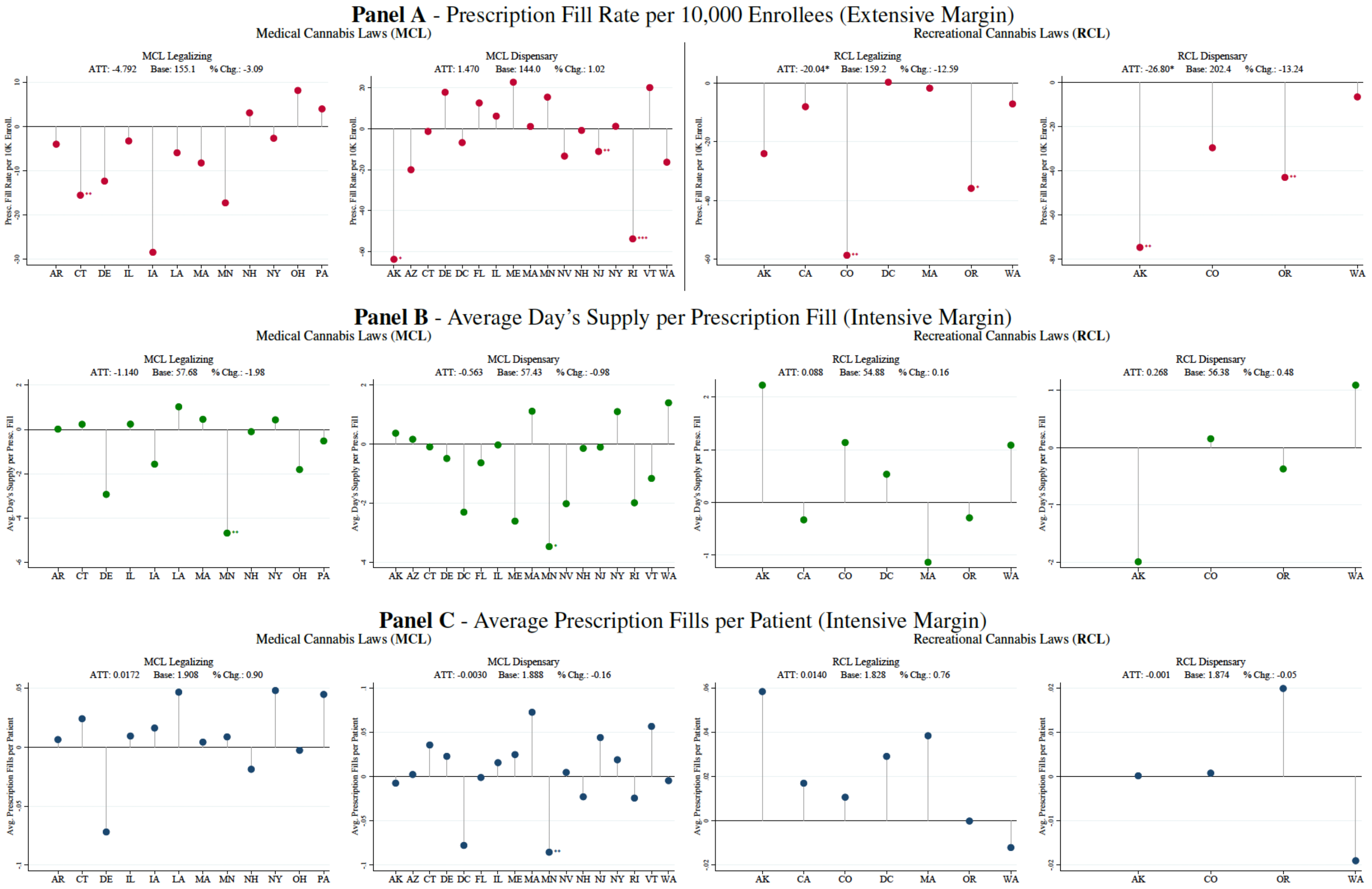

Notes: Authors' calculations using information from Clinformatics® aggregated at the state quarter level. Treatment effects comparing the actual and the synthetic series of outcome variables from individual case studies (Abadie et al. 2010; Abadie 2021). The top of each panel presents the average treatment effect on the treated states, and its comparison to the pre-policy baseline following the permutation inference (Cavallo et al. 2013; Hagemann 2019).

© 2024 Bradford AC et al. *JAMA Network Open.*

eTable 31. Average Treatment Effects Over Sleep Medication Prescription Fills

PANEL A – MEDICAL CANNABIS LAWS

	ATT	Std. Dev.	95% C.I.	p-value	Baseline	%Chg.	+Cases	-Cases	Significant Case Studies
**Prescription Fill Rate per 10,000 Enrollees (Extensive Margin)**									
MCL Legal	-4.792	6.60	[-11.70,9.59]	0.2646	155.1	-3.09	3	9	CT
Dispensary Open	1.470	9.86	[-17.02,15.28]	0.3936	144.0	1.02	8	9	AK(-), NJ(-), RI(-)
**Average Days' Supply per Prescription Fill (Intensive Margin)**									
MCL Legal	-1.140	1.29	[-2.38,1.87]	0.2130	57.68	-1.98	6	6	MN
Dispensary Open	-0.563	1.66	[-3.21,2.20]	0.4092	57.43	-0.98	5	12	MN
**Average Prescription Fills per Patient (Intensive Margin)**									
MCL Legal	0.0172	0.04	[-0.06,0.06]	0.3062	1.908	0.90	9	3	
Dispensary Open	-0.0030	0.05	[-0.07,0.07]	0.4718	1.888	-0.16	10	7	MN

PANEL B – RECREATIONAL CANNABIS LAWS

	ATT	Std. Dev.	95% C.I.	p-value	Baseline	%Chg.	+Cases	-Cases	Significant Case Studies
**Prescription Fill Rate per 10,000 Enrollees (Extensive Margin)**									
RCL Legal	-20.037*	14.59	[-24.77,21.22]	0.0866	159.2	-12.59	1	6	CO, OR
Dispensary Open	-26.800*	19.80	[-41.39,23.85]	0.0970	202.4	-13.24	0	4	AK, OR
**Average Days' Supply per Prescription Fill (Intensive Margin)**									
RCL Legal	0.088	1.42	[-2.56,2.06]	0.4440	54.88	0.16	4	3	
Dispensary Open	0.268	2.41	[-4.13,3.83]	0.4682	56.38	0.48	2	2	
**Average Prescription Fills per Patient (Intensive Margin)**									
RCL Legal	0.0140	0.04	[-0.07,0.07]	0.3242	1.828	0.76	6	1	
Dispensary Open	-0.0009	0.08	[-0.14,0.14]	0.5026	1.874	-0.05	3	1	

© 2024 Bradford AC et al. *JAMA Network Open.*

eFigure 12. Sleep Medication Prescription Fills- Case Study Average Treatment Effect

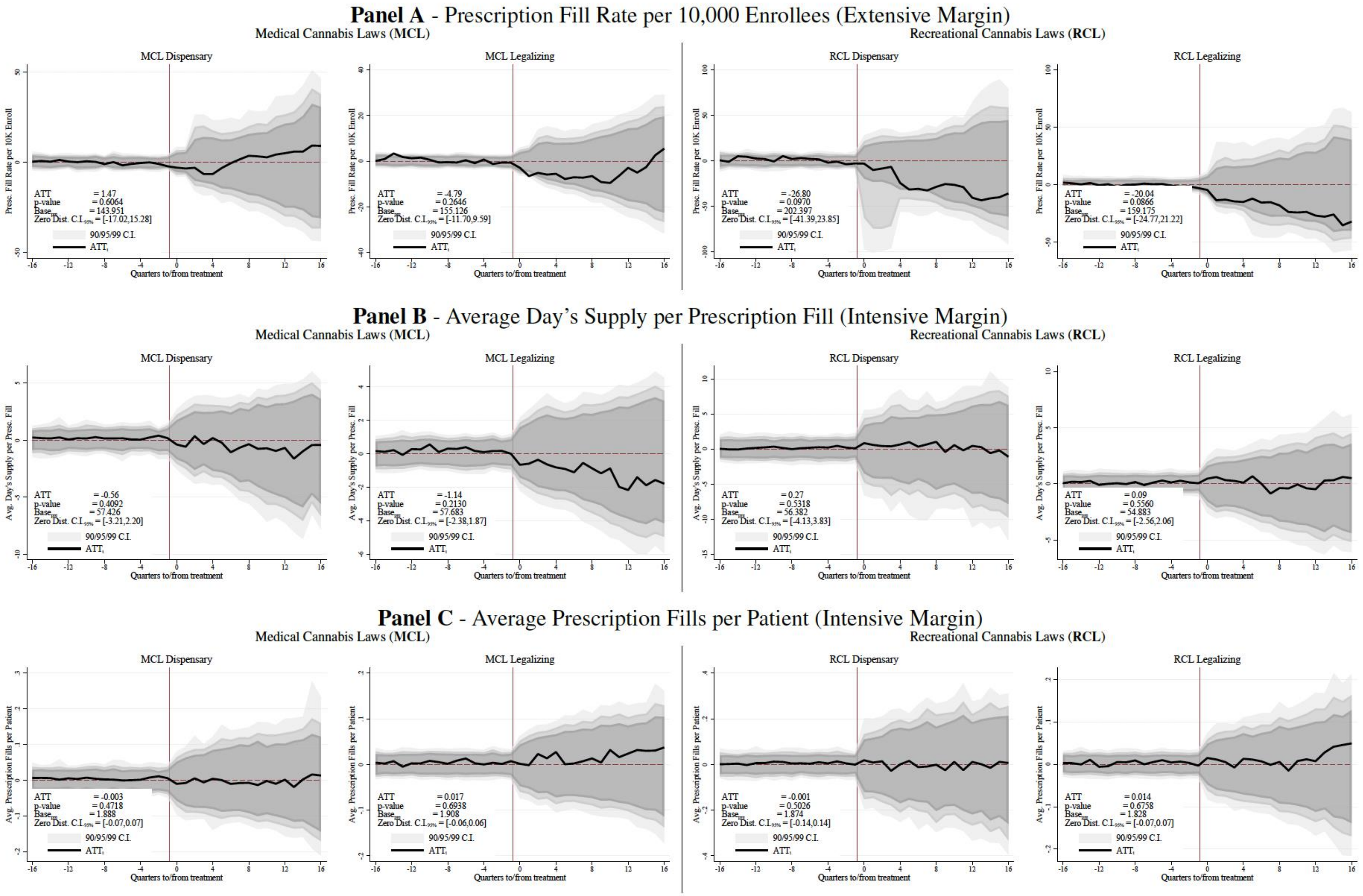

© 2024 Bradford AC et al. *JAMA Network Open.*
