# Supplementary material for: Cannabis Laws and Utilization of Medications for the Treatment of Mental Health Disorders
Source: JAMA Netw Open. 2024 Sep 5;7(9):e2432021. doi: 10.1001/jamanetworkopen.2024.32021 (PMC11377998; doi:10.1001/jamanetworkopen.2024.32021)
Supplement: Supplement 2. — Data Sharing Statement [file jamanetwopen-e2432021-s002.pdf]

Data Sharing Statement

Bradford. Cannabis Laws and Utilization of Medications for the Treatment of Mental Health Disorders. *JAMA Netw Open*. Published September 05, 2024. doi:10.1001/jamanetworkopen.2024.32021

Data
Data available: No

Additional Information
Explanation for why data not available: DUA protected data
